# Ultra-wide-band millimeter-wave generator using spin torque oscillator with strong interlayer exchange couplings

**DOI:** 10.1038/s41598-022-15014-y

**Published:** 2022-07-19

**Authors:** Yuichiro Kurokawa, Keisuke Yamada, Tomohiro Taniguchi, Shu Horiike, Terumitsu Tanaka, Hiromi Yuasa

**Affiliations:** 1 Graduate School and Faculty of Information Science and Electrical Engineering, Kyushu University, Fukuoka, 819-0395 Japan; 2 Department of Chemistry and Biomolecular Science, Faculty of Engineering, Gifu University, Gifu, 501-1193 Japan; 3 Research Center for Emerging Computing Technologies, National Institute of Advanced Industrial Science and Technology (AIST), Tsukuba, Ibaraki 305-8568 Japan

**Keywords:** Magnetic properties and materials, Magnetic devices, Magnetic properties and materials

## Abstract

Recent increased development interest in millimeter-wave oscillator devices has necessitated realization of small oscillators with high frequency, wide frequency tunability, and room-temperature operation. Spin-torque oscillators (STOs) are fascinating candidates for such applications because of their nanometer size and suitability for room-temperature operation. However, their oscillation frequency and tunable range are limited to the order of 100 MHz–10 GHz. Here, we propose use of bilinear (*J*_1_) and biquadratic (*J*_2_) interlayer exchange couplings between ferromagnets in STOs to overcome these problems. The bilinear coupling contributes to oscillation frequency enhancement, whereas the biquadratic coupling facilitates frequency tunability via a current. Using micromagnetic simulation with parameters estimated from a material with small saturation magnetization, for *J*_1_ = 0 and *J*_2_ =  − 1.0 mJ/m^2^, respectively, we find that the STO exhibits high frequency from 23 to 576 GHz and that its tunability reaches 61 GHz/(10^11^ A/m^2^) for current densities of − 0.5 to − 9.5 × 10^11^ A/m^2^. An analytical theory based on the macrospin model is also developed, which exhibits good quantitative agreement with the micromagnetic simulations. These results introduce new possibilities for spintronics applications in high-frequency devices such as next-generation mobile communications.

## Introduction

Millimeter-wave oscillators are attracting considerable attention because of their applicability to telecommunication and sensing devices, such as 5th- and 6th-generation mobile systems and automotive radar^1–6^. Several types of oscillator have been proposed for these applications, such as Gunn diodes^7^, tunnel junction transit time (TUNNETT) diodes^8^, free electron lasers (FELs)^9^, and Ge lasers^10^. However, none of these devices satisfy all practical requirements simultaneously. For example, the oscillation frequencies of the Gunn diode and TUNNETT are fixed, but frequency tunability is necessary for their application in automotive radar^5^. In contrast, both FELs and Ge lasers have frequency tunability; however, the former are of large size owing to their mechanical components and the latter operate at low temperatures only. Thus, realization of millimeter-wave oscillators with small size, frequency tunability, and room-temperature operation is highly desirable.

The spin torque oscillator (STO) is a fascinating candidate for realizing a millimeter-wave oscillator. STOs consist of a ferromagnetic (FM)/nonmagnetic (NM)/FM trilayer on the nanometer scale and operate at room temperature. The electric current injected into an STO induces magnetization oscillation via the spin-transfer effect, which can be electrically detected through the magnetoresistance effect. Magnetic anisotropy in the ferromagnet enables oscillation frequency $f$ tuning through manipulation of the magnitude of the electric current^11–20^. Therefore, STOs satisfy several requirements for millimeter-wave oscillator applications. However, its oscillation frequency and tunable range are typically limited to the order of 100 MHz–10 GHz^11–24^, where the vortex oscillators show a relatively low frequency^17,21–23^, while an STO under a large applied field shows an oscillation frequency of approximately 60 GHz^24^. From the viewpoint of theoretical study, the millimeter-wave STO using an antiferromagnetic material with small size, frequency tunability, and room-temperature operation has been reported^25,26^. However, its operation current density is too large to utilize it (> 10^12^ A/m^2^). Theoretically, the frequency can be enhanced by increasing the magnitude of the magnetic field. Therefore, this limitation can be overcome by external magnetic field. However, the magnitude of the magnetic field that corresponds to an oscillation frequency of 100 GHz is approximately 3.5 T. An internal magnetic field can be used instead of an external magnetic field. For example, S. Tsunegi, et al. have been reported a spin torque diode effect^27^, where an oscillating current induces a magnetization oscillation and thus, it is an inverse effect of a self-oscillation in an STO. There, a resonance of the magnetization with an oscillation frequency of approximately 70 GHz was observed due to a large magnetic anisotropy. Even in this case, however, an external magnetic field was still required to achieve such a high frequency. A circuit generating such a high magnetic field will considerably increase the size of the whole device making it impractical. It is necessary to be able to increase the oscillation frequency to the order of 300 GHz without requiring an external magnetic field for applying STOs to millimeter-wave oscillator devices.

In this work, we propose an STO with strong bilinear and biquadratic interlayer exchange couplings between the free and reference FM layers. Although bilinear interlayer exchange coupling has been employed in several spintronics devices^28–31^ and its role in magnetization oscillation has been partially studied to date^32,33^, the role of the biquadratic interlayer exchange coupling^34–42^ has not been investigated in detail. Here, we find that the bilinear interlayer exchange coupling contributes to oscillation frequency enhancement, whereas the biquadratic interlayer exchange coupling facilitates frequency tunability. Through micromagnetic simulations based on the Landau–Lifshitz–Gilbert (LLG) equation, we find that the oscillation frequency can be tuned in the range of 23 to 576 GHz, and that its tunability reaches 61 GHz/(10^11^ A/m^2^). These values indicate the applicability of the present STO to millimeter-wave oscillators.

## Results

### Model definition

**Figure 1:**
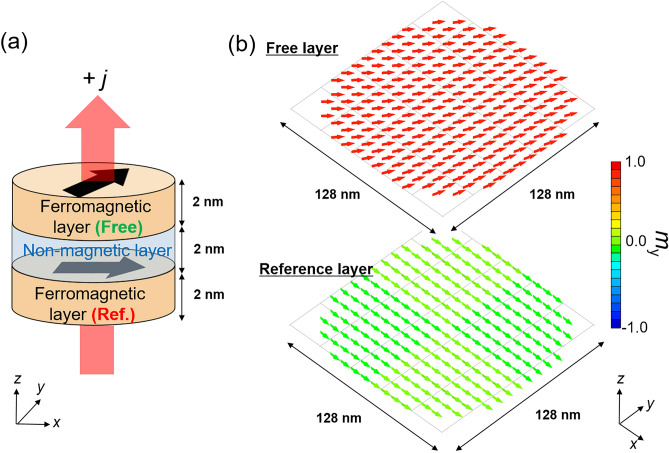
(**a**) Schematic illustration of FM/NM/FM trilayer. (**b**) Equilibrium magnetization state in trilayer magnet with $\left({J}_{1},{J}_{2}\right)=(0.6,-0.6)$ mJ/m^2^. The arrows indicate the magnetic moment directions and the colors indicate the *y*-direction magnetization components. The arrows were enlarged using average magnetic vector of $4\times 4$ square prisms.

Here, we provide a model of an STO consisting of a cylinder-shaped FM/NM/FM metallic trilayer, as schematically shown in Fig. 1a. The bottom and top FMs correspond to the reference and free layers, respectively. By applying an electric current in the *z*-direction perpendicular to the film plane, a spin-transfer torque is excited on the magnetization in the free layer and magnetization oscillation is induced. In this study, the magnetization dynamics are investigated using micromagnetic simulations based on the LLG equation. For convenience, we use subscripts $i,k=1, 2$ $(i\ne k)$ to distinguish the quantities of the free $(i=1)$ and reference $(i=2)$ layers. In addition, we use subscripts $x, y, z$ to distinguish the spatial components of the magnetization. Accordingly, the unit vector pointing in the magnetization direction is denoted as ${\mathbf{m}}_{i}$, and its components are denoted as ${m}_{i,x}$. As we perform a micromagnetic simulation, ${\mathbf{m}}_{i}$ depends on the position in the FM. Throughout this work, we assume that the thickness and diameter of both the free and reference layers are 2 and 128 nm, respectively, and that the NM thickness is also 2 nm (see Fig. 1). The LLG equation for ${\mathbf{m}}_{i}$ incorporates the torque due to the effective magnetic field ${\mathbf{H}}_{\mathrm{eff}}$, the damping torque, and the spin-transfer torque, and is given by^43–46^

1 $$ \frac{{d{\mathbf{m}}_{i} }}{dt} = - \gamma \left( {{\mathbf{m}}_{i} \times {\mathbf{H}}_{{{\text{eff}}}} } \right) + \alpha_{i} \left( {{\mathbf{m}}_{i} \times \frac{{d{\mathbf{m}}_{i} }}{dt}} \right) - \frac{{g\mu_{{\text{B}}} jp}}{{2eM_{i} d_{i} }}{\mathbf{m}}_{i} \times \left( {{\mathbf{m}}_{2} \times {\mathbf{m}}_{1} } \right), $$

where $\gamma$, ${\alpha }_{i}$, $g$, ${M}_{i}$, ${d}_{i}$, and $p$ are the gyromagnetic constant, Gilbert damping constant, Lande *g* factor, saturation magnetization, thickness of the FM, and spin polarization, respectively. The current density is denoted as $j$, where a positive current corresponds to electrons flowing from the free to the reference layer; that is, the positive current moves the magnetization in the free layer antiparallel to that in the reference layer. The Bohr magneton and elementary charge are ${\mu }_{\mathrm{B}}$ and $e (>0)$, respectively. The details of the material parameters and so on are summarized in “Methods”.

The effective magnetic field ${\mathbf{H}}_{\mathrm{eff}}$ is given by

2 $$ {\mathbf{H}}_{{{\text{eff}}}} = {\mathbf{H}}_{{{\text{ex}}}} + {\mathbf{H}}_{{{\text{st}}}} + {\mathbf{H}}_{{{\text{bl}}}} + {\mathbf{H}}_{{{\text{bq}}}} , $$

where ${\mathbf{H}}_{\mathrm{ex}}$ and ${\mathbf{H}}_{\mathrm{st}}$ are the exchange fields between the local magnetic moments and the static (magnetic dipolar) field, respectively. The effective magnetic fields ${\mathbf{H}}_{\mathrm{bl}}$ and ${\mathbf{H}}_{\mathrm{bq}}$ originate from the interlayer exchange couplings between the free and reference layers, the details of which are given below. In addition to Eq. (2), a pinning magnetic field of ${{\mu }_{0}H}_{\mathrm{pin}}^{\mathrm{R}}=500$ mT is applied to the reference layer in the positive *x*-direction^47^. In STOs, the pinning field originates from the exchange bias from a pinned layer, the magnetization direction of which is fixed through attachment of an antiferromagnetic layer composed of a material such as IrMn^42^. Because of the large pinning field, the reference-layer magnetization is approximately fixed in the positive *x*-direction.

The material parameters of the reference layer used in the present work are derived from an experimental investigation of CoFe^42^, which is frequently used in typical STOs (see detailed in “Methods” as the simulations). In addition, we assume that the free layer consists of a material with small saturation magnetization, for example, NiCu alloy^48^, because this small saturation magnetization generates large interlayer exchange coupling fields and enhances the oscillation frequency, as discussed in detail below. While the damping constant of NiCu has not been extensively investigated, we assume that it is close to that of pure nickel^49^.

### Interlayer exchange couplings

The key magnetic property in this work is the interlayer exchange coupling between the free and reference layers. The interlayer exchange coupling has two contributions: the bilinear and biquadratic couplings. The bilinear interlayer exchange coupling is attributed to the Ruderman–Kittel–Kasuya–Yoshida (RKKY) interaction carried by the itinerant electrons between two FMs and changes its magnitude and sign according to variations in the NM thickness^50–53^. The bilinear coupling prefers ferromagnetic (antiferromagnetic) alignment of the magnetization when the coupling constant ${J}_{1}$ has a positive (negative) sign. The biquadratic coupling originates from the FM/NM/FM band structure^36^, spatial fluctuations of the bilinear coupling due to terraced variations in the NM thickness^34^, and the localized atomic-electron state in the NM called loose spin^35^. For example, biquadratic coupling has been observed for CoFe/ion-assisted oxidation of CoFe/CoFe spin valves^37^.

The biquadratic coupling energy for the positive coupling constant ${J}_{2}$ is minimized when the magnetization alignment is either ferromagnetic or antiferromagnetic, whereas an orthogonal alignment minimizes the biquadratic coupling energy when ${J}_{2}$ is negative. Accordingly, the interlayer exchange couplings provide the following energy per unit area^35^:

3 $$ E_{{\text{c}}} = - J_{1} \cos \theta - J_{2} \cos^{2} \theta , $$

where $\theta ={\mathrm{cos}}^{-1}{\mathbf{m}}_{1}\bullet {\mathbf{m}}_{2}$ is the angle between the magnetizations in the two FMs. The typical value of ${J}_{1}$ for an Fe/Cr superlattice is of the order of 1.0 mJ/m^2^^28^, whereas that of ${J}_{2}$ is − 1.85 mJ/m^2^ for a Co_2_MnSi/Cr/Co_2_MnSi trilayer^38^. We assume that the values of ${J}_{1}$ and ${J}_{2}$ in the present system are of the same order, although the values in CoFe/NM/NiCu have not been reported.

The interlayer exchange couplings provide effective magnetic fields ${\mathbf{H}}_{\mathrm{bl}}$ and ${\mathbf{H}}_{\mathrm{bq}}$ in Eq. (2). The effective magnetic fields ${\mathbf{H}}_{\mathrm{bl}}$ and ${\mathbf{H}}_{\mathrm{bq}}$ associated with the bilinear and biquadratic couplings are given by^54^

4 $$ { }{\mathbf{H}}_{{{\text{bl}},i}} = \frac{{J_{1} }}{{\mu_{0} M_{i} d_{i} }}{\mathbf{m}}_{k} , $$

5 $$ {\mathbf{H}}_{{{\text{bq}},i}} = \frac{{2J_{2} }}{{\mu_{0} M_{i} d_{i} }}\left[ {\begin{array}{*{20}c} {m_{k,x} \left( {m_{i,x} m_{k,x} + m_{i,y} m_{k,y} + m_{i,z} m_{k,z} } \right)} \\ {m_{k,y} \left( {m_{i,x} m_{k,x} + m_{i,y} m_{k,y} + m_{i,z} m_{k,z} } \right)} \\ {m_{k,z} \left( {m_{i,x} m_{k,x} + m_{i,y} m_{k,y} + m_{i,z} m_{k,z} } \right)} \\ \end{array} } \right]. $$

We next explain the influence of the effective magnetic fields ${\mathbf{H}}_{\mathrm{bl}}$ and ${\mathbf{H}}_{\mathrm{bq}}$ on the magnetization oscillation in the free layer. As mentioned above, we can assume that the magnetization in the reference layer is approximately fixed in the positive *x*-direction. For convenience, we assume that *J*_1_ and *J*_2_ are positive and negative, respectively. In this case, ${\mathbf{H}}_{\mathrm{bl},1}\cong \left[{J}_{1}/\left({\mu }_{0}{M}_{1}{d}_{1}\right)\right]\widehat{\mathbf{x}}$ and ${\mathbf{H}}_{\mathrm{bq},1}\cong -\left[2|{J}_{2}|/\left({{\mu }_{0}M}_{1}{d}_{1}\right)\right]{m}_{1,x}\widehat{\mathbf{x}}$ play the same roles as an external magnetic field and the shape magnetic anisotropy field in the *x*-direction, respectively. Therefore, the former induces magnetization oscillation with constant frequency ${f}_{1}=\left[{\gamma J}_{1}/\left(2\pi {M}_{1}{d}_{1}\right)\right]$. The latter contribution yields the oscillation frequency ${f}_{2}=\left[{2\gamma J}_{2}/\left(2\pi {M}_{1}{d}_{1}\right)\right]{m}_{1,x}$, which depends on the oscillation amplitude through ${m}_{1,x}$. Therefore, the bilinear interlayer exchange coupling contributes to oscillation frequency enhancement, whereas the biquadratic interlayer exchange coupling provides frequency tunability. Hence, millimeter-wave frequency and wide tunability are simultaneously achieved.

To enhance the effect of the interlayer exchange couplings on the magnetization oscillation, we assume small saturation magnetization in the free layer, as mentioned above. This is because the effective fields associated with the interlayer exchange couplings are inversely proportional to saturation magnetization, as detailed in Eqs. (4) and (5). Figure 1b shows sample equilibrium magnetization alignments in two FMs in the absence of current, for ${J}_{1}$ and ${J}_{2}$ of 0.6 and − 0.6 mJ/m^2^, respectively^42^. The average relative angle between the two FMs is approximately ${60}^{^\circ }$. Note that this value is close to the angle ${\mathrm{cos}}^{-1}\left[-{J}_{1}/{(2J}_{2})\right]={60}^{^\circ }$, which minimizes the interlayer exchange coupling energy given by Eq. (3). This result indicates that the ${\mathbf{H}}_{\mathrm{bl}}$ and ${\mathbf{H}}_{\mathrm{bq}}$ associated with the interlayer exchange couplings dominantly determine the magnetization direction, although other factors, such as ${\mathbf{H}}_{\mathrm{st}}$, affect the relative angle between the magnetizations.

### Micromagnetic simulation results

Here, we describe the magnetization dynamics in the free layer, as determined from micromagnetic simulations. For convenience, we focus on the negative current region. The dynamics in the positive current region is discussed in Supplementary Information.

**Figure 2:**
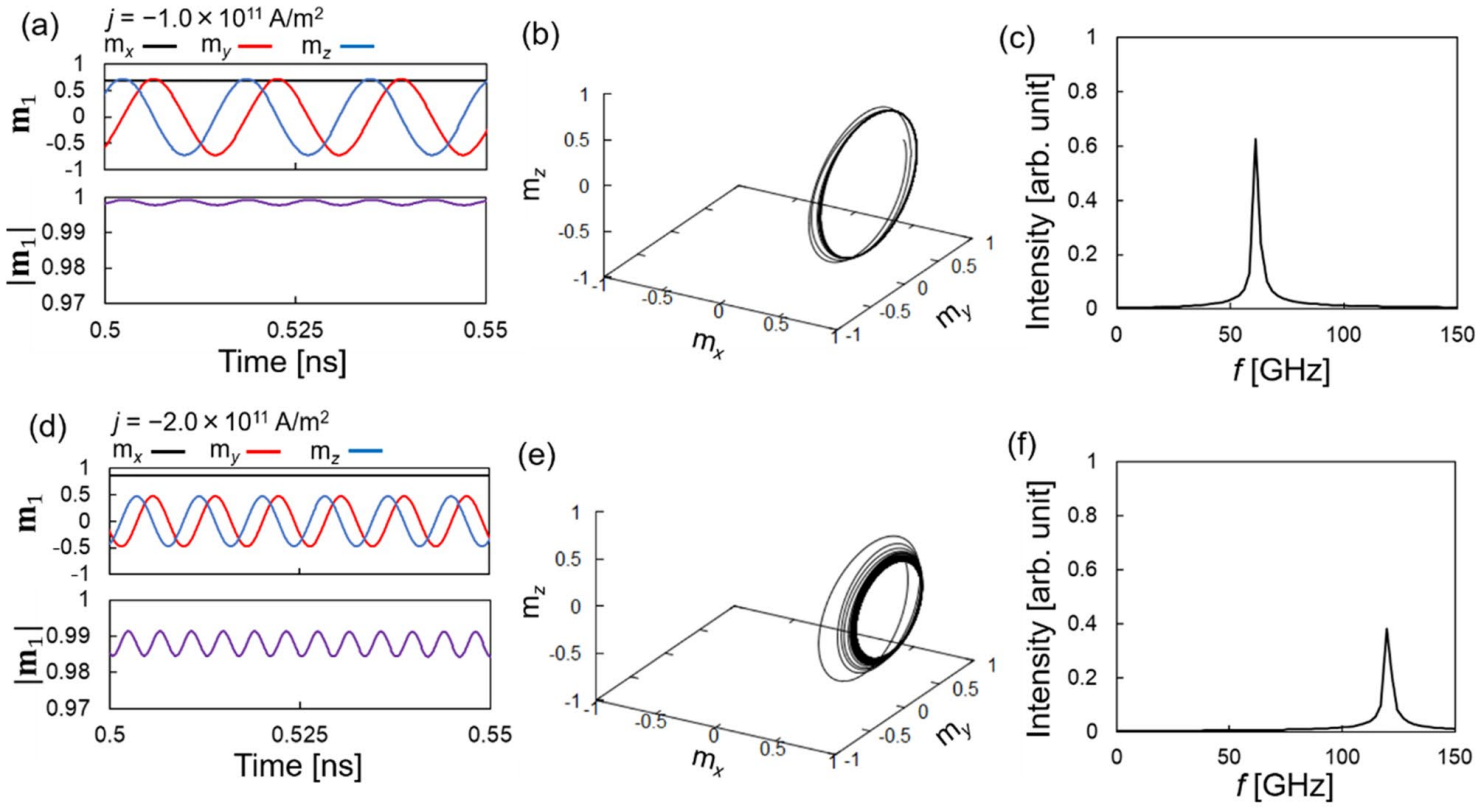
(**a**, **d**) Time evolution of averaged ${\mathbf{m}}_{1}$ and $|{\mathbf{m}}_{1}|$, (**b**, **e**) ${\mathbf{m}}_{1}$ trajectories, and (**c**, **f**) Fourier spectra of ${m}_{1,y}$ for $j=-1.0\times {10}^{11}$ and $-2.0\times {10}^{11}$ A/m^2^, respectively. The interlayer exchange couplings are $\left({J}_{1},{J}_{2}\right)=(0.6,-0.6)$ mJ/m^2^.

As an example, we show the magnetization dynamics for fixed values of the interlayer exchange couplings for ${J}_{1}=0.6$ mJ/m^2^ and ${J}_{2}=-0.6$ mJ/m^2^. A visible oscillation is excited by a current density with magnitude $|j|$ exceeding $j=-0.25\times {10}^{11}$ A/m^2^. The minimum current density considered in the simulation is $j=-0.1\times {10}^{11}$ A/m^2^. First, we discuss the dynamic behavior for a relatively small current. Figure 2a,b show the time evolutions of the ${\mathbf{m}}_{1}$ components averaged over the sites and the spatial trajectory obtained for $j=-1.0\times {10}^{11}$ A/m^2^, respectively. The magnitude of the averaged ${\mathbf{m}}_{1}$, $\left|{\mathbf{m}}_{1}\right|=\sqrt{{\langle {m}_{1,x}\rangle }^{2}+{\langle {m}_{1,y}\rangle }^{2}+{\langle {m}_{1,z}\rangle }^{2}}$, which is close to one, is also shown. Note that the LLG equation conserves the magnitude of the magnetic moment at each site. However, the magnitude of the averaged ${\mathbf{m}}_{1}$ is conserved only when all magnetic moments move uniformly. The result shown in Fig. 2a indicates excitation of an approximately uniform oscillation of the magnetization around the *x*-axis, where ${m}_{1,x}$ is almost constant. For ${m}_{1,y}$ and ${m}_{1,z}$, approximately the same oscillation amplitudes appear. We emphasize that such oscillations are mainly induced by the interlayer exchange couplings, for the following reason.

In existing STOs^11,12,16^, the magnetization oscillation amplitude is usually suppressed in the *z*-direction owing to a large demagnetization (shape magnetic anisotropy) field. In the present STO, however, the demagnetization field ${{\mu }_{0}M}_{1}\cong 0.06$ T, which is far smaller than the ${{\mu }_{0}\mathbf{H}}_{\mathrm{bl}}$ and ${{\mu }_{0}\mathbf{H}}_{\mathrm{bq}}$ magnitudes given by ${\mu }_{0}\left[{J}_{1}/\left({{\mu }_{0}M}_{1}{d}_{1}\right)\right]=6$ T and ${\mu }_{0}\left[2|{J}_{2}|/\left({\mu }_{0}{M}_{1}{d}_{1}\right)\right]=12$ T. The large difference between the demagnetization field and the effective magnetic fields associated with the interlayer exchange couplings originates from the small saturation magnetization ${M}_{1}$ in the free layer, as the former and latter are proportional and inversely proportional to ${M}_{1}$, respectively. Accordingly, the effect of the shape magnetic anisotropy on the magnetization dynamics is relatively small, and the dynamic trajectory is mainly determined by the torque due to the fields associated with the interlayer exchange couplings. Because the magnetization in the reference layer is approximately fixed in the *x*-direction, both ${\mathbf{H}}_{\mathrm{bl}}$ and ${\mathbf{H}}_{\mathrm{bq}}$ have axial symmetries around the *x-*axis. Hence, magnetization oscillation with an approximately common oscillation amplitude in the *y*-and *z-*directions is excited around the *x-*axis.

Figure 2c shows the Fourier spectrum of ${m}_{1,y}$ for $j=-1.0\times {10}^{11}$ A/m^2^, for which the peak frequency appears at 61 GHz. We emphasize that this peak frequency is significantly larger than those found in in-plane magnetized STOs^11,12,16^, which are typically of the order of 1 GHz. A high frequency originates from the large interlayer exchange couplings. In this study, we evaluated the peak frequency by varying $j$ over the range of $-2.5\times {10}^{11}\le j\le -0.5\times {10}^{11}$ A/m^2^; hence, the tunability was found to be 63 GHz/(10^11^ A/m^2^).

We also investigated the dynamic behavior for relatively large current density. Figure 2d,e show the time evolutions of the averaged components of ${\mathbf{m}}_{1}$ and their magnitudes, and the spatial trajectory obtained for $j=-2.0\times {10}^{11}$ A/m^2^, respectively. Similar to the small-current region, we obtained an approximately uniform magnetization oscillation; see also, videos in the Supplementary Information. Further, $f$ reached 120 GHz, as estimated from the Fourier spectrum shown in Fig. 2f.

**Figure 3:**
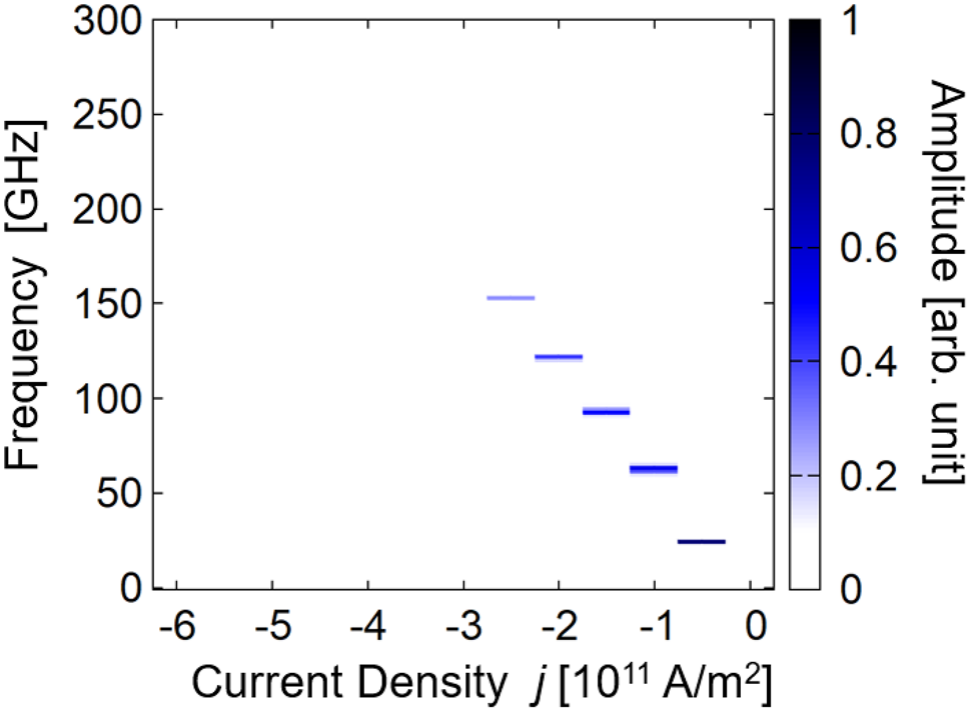
Dependence of $f$ on $j$ estimated from Fourier spectrum for $\left({J}_{1},{J}_{2}\right)=(0.6,-0.6)$ mJ/m^2^. The Fourier spectrum amplitude is indicated by the color gradient.

Figure 3 summarizes the oscillation frequency and Fourier spectrum amplitude dependence on current density. Linear dependence of the frequency at the amplitude peak position on current density is apparent; this is evidence of the excitation of macrospin-like oscillation, as explained below. Notably, oscillation frequency and its tunability reach 152 GHz and 63 GHz/(10^11^A/m^2^), respectively; these estimated values are considerably higher than those of conventional FM layers without bilinear and biquadratic couplings described in previous reports^11,12,16^.

### Oscillation frequency for various J_1_ and J_2_

**Figure 4:**
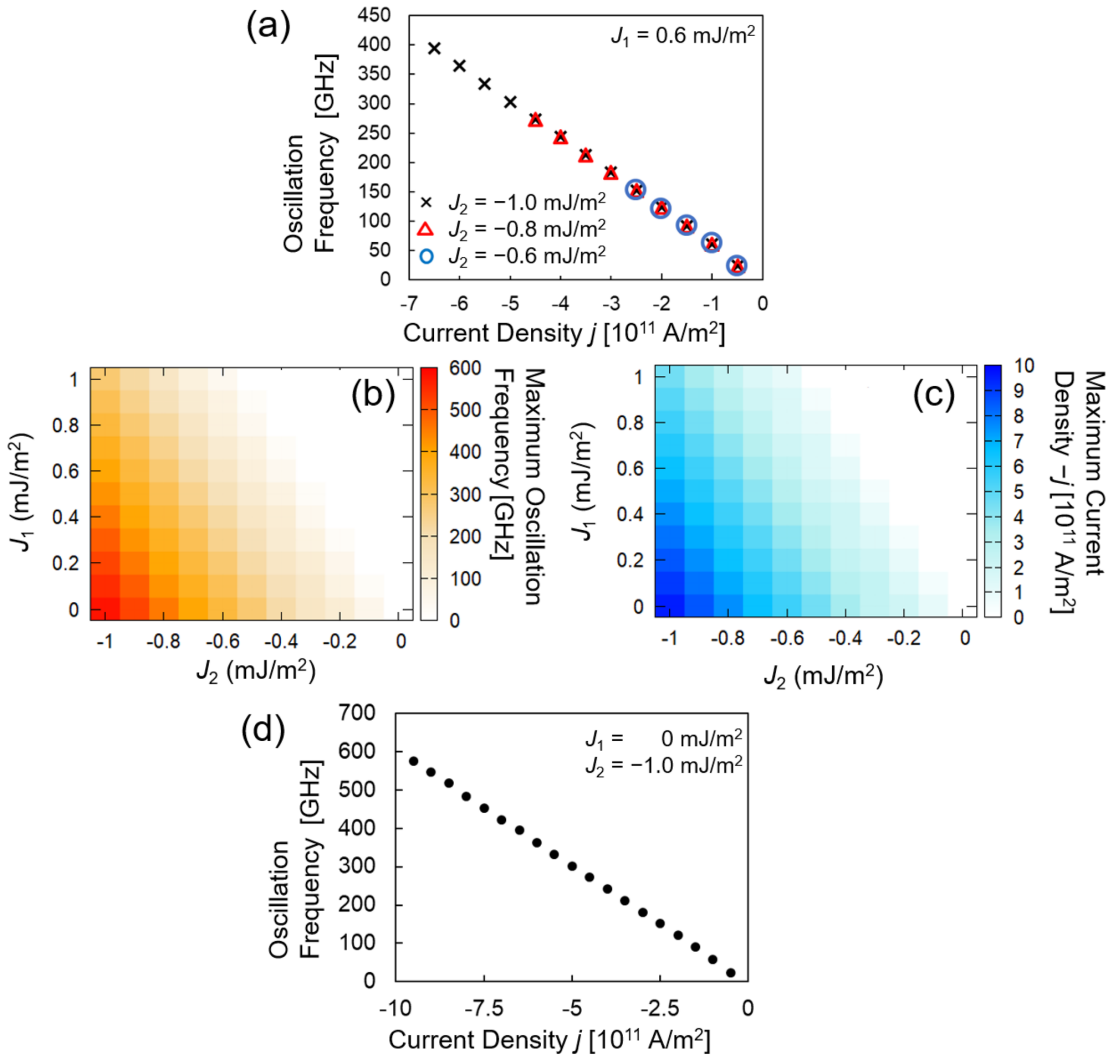
(**a**) Plot of $f$ as function of *j* for ${J}_{2}=$ −0.6, − 0.8, and − 1.0 mJ/m^2^ with ${J}_{1}$ fixed to 0.6 mJ/m^2^. Dependence of maximum (**b**) $f$ and (**c**) $j$ realizing oscillation on ${J}_{1}$ and ${J}_{2}$. We determined that the magnetization is in a non-oscillating state when the maximum value of the normalized Fourier spectrum is less than 10^–2^. (**d**) Plot of $f$ as function of *j* for $\left({J}_{1},{J}_{2}\right)=(0,-1.0)$ mJ/m^2^.

Figure 4a shows the oscillation frequency dependence on current density, where the strength of the bilinear interlayer exchange coupling is fixed to ${J}_{1}=0.6$ mJ/m^2^ and that of the biquadratic interlayer exchange coupling is varied, i.e., ${J}_{2}=-0.6, -0.8, \mathrm{and}-1.0$ mJ/m^2^. We first note that the oscillation frequency in a relatively small current-magnitude region is approximately independent of ${J}_{2}$. However, the current–density range for which the oscillation is observed depends on ${J}_{2}$. For example, oscillation is observed for $-2.5\times {10}^{11}\le j\le -0.5\times {10}^{11}$ A/m^2^ when ${J}_{2}=-0.6$ mJ/m^2^, but for $-4.5\times {10}^{11}\le j\le -0.5\times {10}^{11}$ A/m^2^ when ${J}_{2}=-0.8$ mJ/m^2^. The mechanism underlying this result is explained by the analytical theory presented in “Discussion”.

We performed similar simulations in which ${J}_{1}$ was varied. Figure 4b summarizes the dependence of the maximum oscillation frequency on ${J}_{1}$ and ${J}_{2}$, and Fig. 4c shows the maximum current density required to excite the oscillation. It is notable that ultra-wide frequency tunability of 23–576 GHz was obtained at $\left({J}_{1},{J}_{2}\right)=\left(0, -1.0\right)$ mJ/m^2^ and $-9.5\times {10}^{11} \le j\le -0.5\times {10}^{11}\mathrm{A}/{\mathrm{m}}^{2}$. Additionally, its tunability reaches 61 GHz/(10^11^A/m^2^); see Fig. 4d. In the white-colored region in Fig. 4b, the free-layer magnetization does not oscillate; there, the bilinear interlayer exchange coupling, which is larger than its biquadratic counterpart, fixes the magnetization direction parallel to the *x*-direction.

Figure 4c indicates that the maximum current density, which is just below a current density when the spin torque oscillation is stopped, increases with increasing $|{J}_{2}|$. This is because the effective magnetic field associated with the biquadratic coupling, ${\mathbf{H}}_{\mathrm{bq},1}\cong -\left[2|{J}_{2}|/\left({\mu }_{0}{M}_{1}{d}_{1}\right)\right]{m}_{1,x}\widehat{\mathbf{x}}$, determines the maximum current density. ${\mathbf{H}}_{\mathrm{bq},1}$ plays the same role as the demagnetization field in the *x*-direction. Therefore, the biquadratic interlayer exchange coupling prefers the free-layer magnetization alignment orthogonal to the *x*-direction because ${\mathbf{H}}_{\mathrm{bq},1}$ makes the free-layer magnetization unstable when the free-layer magnetization is aligned parallel to the *x*-direction. In contrast, the spin-transfer torque moves the free-layer magnetization parallel to the *x*-direction because the spin polarization generated from the reference layer approximately points in that direction. As a result, the damping torque associated with ${\mathbf{H}}_{\mathrm{bq},1}$ and the spin-transfer torque compensate for each other. Note that the constant value of ${m}_{1,x}$ in the oscillation state (Fig. 2a), for example, is determined by this competition. The maximum current density shown in Fig. 4c corresponds to that required to move the free-layer magnetization completely parallel to the *x*-direction, that is, ${m}_{1,x}=1$, by overcoming the damping torque associated with ${\mathbf{H}}_{\mathrm{bq},1}$. When the free-layer magnetization is completely aligned parallel to the *x*-direction, the spin torque oscillation is stopped. Therefore, the maximum current density increases with increasing ${\mathbf{H}}_{\mathrm{bq},1}\propto |{J}_{2}|$.

### Magnetization oscillation for various α and M

The dependences of oscillation frequency on Gilbert damping constant and saturation magnetization in the free layer are discussed here. The interlayer exchange coupling constants are fixed to ${J}_{1}=0.6$ mJ/m^2^ and ${J}_{2}=-0.6$ mJ/m^2^.

**Figure 5:**
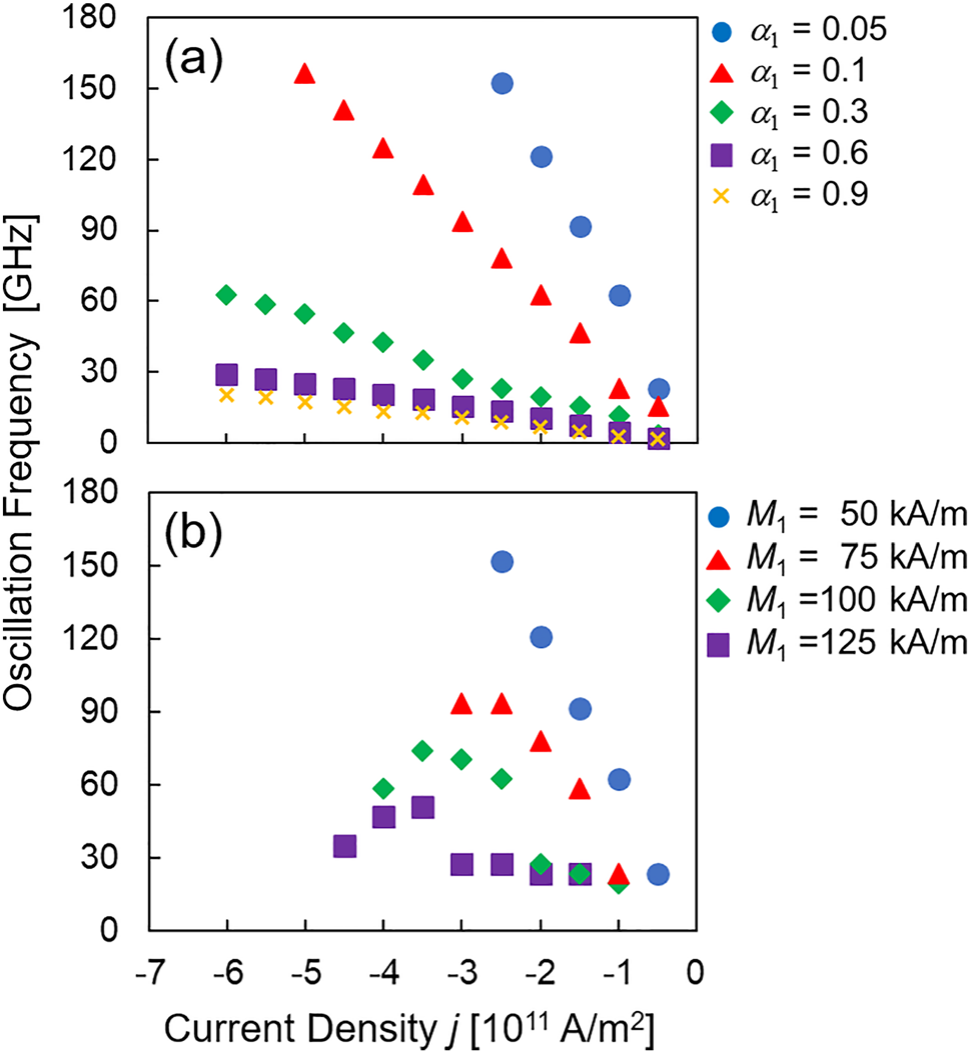
(**a**) Plot of $f$ as function of $j$ for ${\alpha }_{1}=$ 0.05, 0.1, 0.3, 0.6, and 0.9, with ${J}_{1}$, ${J}_{2}$, and ${M}_{1}$ fixed to 0.6 and − 0.6 mJ/m^2^, and 50 kA/m, respectively. (**b**) Plot of $f$ as function of $j$ for ${M}_{1}$ = 50, 75, 100, and 125 kA/m, with ${J}_{1}$, ${J}_{2}$, and ${\alpha }_{1}$ fixed to 0.6 and − 0.6 mJ/m^2^, and 0.05, respectively.

Figure 5a summarizes the relationship between oscillation frequency and current density for various values of ${\alpha }_{1}$. The results indicate that oscillation frequency increases with decreasing damping constant. This is because the spin-transfer torque can easily move the magnetization to a high-frequency state when damping constant is small. The analytical theory presented below also explains this relationship. Figure 5b shows the dependence of the oscillation frequency on the saturation magnetization in the free layer, where a high oscillation frequency is achieved for a small ${M}_{1}$. This is because the ${\mathbf{H}}_{\mathrm{bl}}$ and ${\mathbf{H}}_{\mathrm{bq}}$ associated with the interlayer exchange couplings, which are the dominant contributions to oscillation frequency, are inversely proportional to ${M}_{1}$. The results in Fig. 5 quantitatively indicate that ferromagnetic materials with small ${\alpha }_{1}$ and ${M}_{1}$ are practically preferable, because of their high frequency and wide tunability in the present STO.

## Discussion

Micromagnetic simulation results shown above indicate that the present STO can emit high-frequency signal and have a wide frequency tunability. It was also found that the frequency and its tunability depend on the material parameters such as the saturation magnetization and the damping constant. Here, we discuss the relation between the oscillation frequency and the material parameters from the viewpoint of an analytical theory. We show a formula to explain the relationships between oscillation frequency and the material parameters. Let us assume that the magnetization in the reference layer is fixed in the positive *x-*direction. We retain only ${\mathbf{H}}_{\mathrm{bl}}$ and ${\mathbf{H}}_{\mathrm{bq}}$ in the effective magnetic field because the magnitude of the shape magnetic anisotropy field is far smaller than those of ${\mathbf{H}}_{\mathrm{bl}}$ and ${\mathbf{H}}_{\mathrm{bq}}$. The magnetization oscillation is excited when the spin-transfer torque is balanced with the damping torque^55^. This condition can be written as

6 $$ \alpha_{1} \gamma \left( {h_{{{\text{bl}}}} + h_{{{\text{bq}}}} m_{1,x} } \right) - \frac{{g\mu_{{\text{B}}} jp}}{{2eM_{1} d_{1} }} = 0, $$

where ${h}_{\mathrm{bl}}={J}_{1}/\left({\mu }_{0}{M}_{1}{d}_{1}\right)$ and ${h}_{\mathrm{bq}}=2{J}_{2}/\left({\mu }_{0}{M}_{1}{d}_{1}\right)$. Hence, ${m}_{1,x}$ in the oscillation state is

7 $$ m_{1,x} = - \frac{{h_{{{\text{bl}}}} }}{{h_{{{\text{bq}}}} }} + \frac{{g\mu_{{\text{B}}} jp}}{{2e\alpha_{1} \gamma M_{1} d_{1} h_{{{\text{bq}}}} }}. $$

Note that the oscillation frequency is given by $f=\gamma \left({h}_{\mathrm{bl}}+{h}_{\mathrm{bq}}{m}_{1,x}\right)/\left(2\pi \right)$. Substituting Eq. (7) for this expression, we found that the oscillation frequency can be expressed as

8 $$ f = \frac{{g\mu_{{\text{B}}} p}}{{4\pi \alpha_{1} eM_{1} d_{1} }}j{ }. $$

Equation (8) explains several features revealed by the micromagnetic simulations discussed. First, oscillation frequency linearly increases with increasing current density. Second, oscillation frequency does not explicitly depend on the interlayer exchange coupling. Third, oscillation frequency increases with decreasing damping constant. We also note that Eq. (6) explains the dependence of oscillation frequency on ${J}_{2}$, as observed in Fig. 4a, where the current range necessary to excite the oscillation increases with increasing ${J}_{2}$. Equation (6) indicates that the current density required to balance the spin-transfer torque and damping torque increases linearly with increasing ${J}_{2}$. In fact, from the ${m}_{1,x}$ given by Eq. (7), ${m}_{1,x}\propto j/{J}_{2}$. Note that ${|m}_{1,x}|\le 1$. Accordingly, the range of the current density satisfying the condition ${|m}_{1,x}|\le 1$ can become large with increasing ${J}_{2}$. Therefore, although the oscillation frequency given by Eq. (8) does not explicitly depend on ${J}_{2}$, the interlayer exchange coupling determines the current density range corresponding to the oscillation, as shown in Fig. 4a.

**Figure 6:**
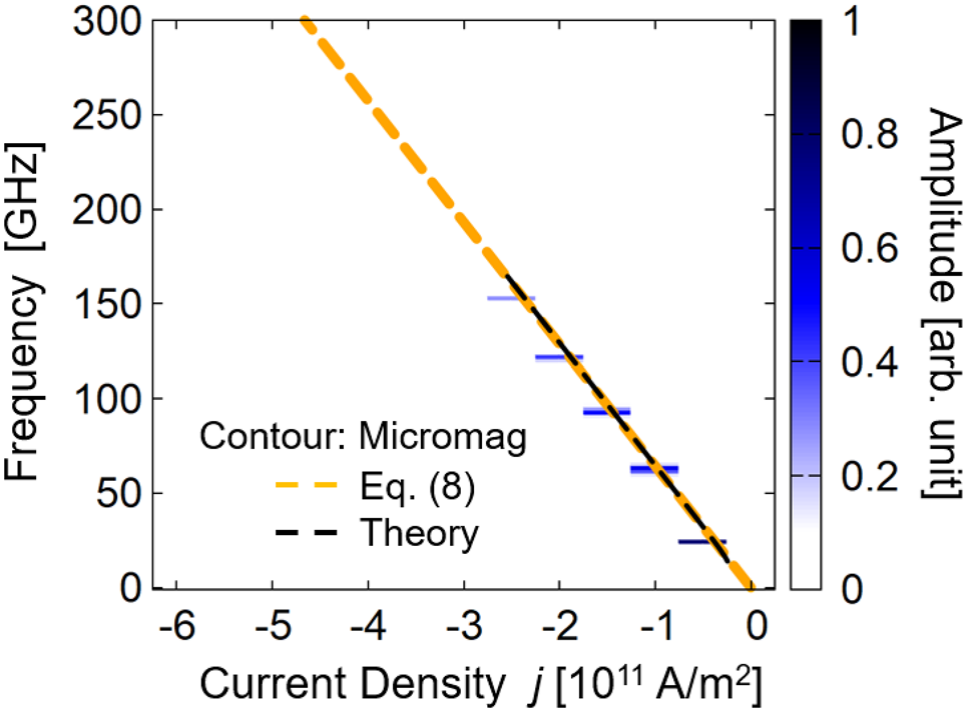
Current dependence of Fourier spectrum (contour), $f$ estimated from Eq. (8) (orange broken lines), and analysis including magnetic anisotropies (black broken lines), with ${J}_{1}=$ 0.6 mJ/m^2^ and ${J}_{2}=$ −0.6 mJ/m^2^.

The contour and orange broken lines in Fig. 6 show the relationship between current density and oscillation frequency estimated from the micromagnetic simulation and Eq. (8), respectively. Quantitatively good agreement between the micromagnetic simulation and analytical theory is obtained in the negative-current region, indicating the validity of the above theory.

However, a small discrepancy exists between the analytical solution of Eq. (8) and the numerical simulation results. In particular, in the low-current–density region, the micromagnetic simulation indicates that a finite current is necessary for oscillation but Eq. (8) predicts that an infinitesimal current can excite an oscillation. Strictly speaking, a finite current density is necessary to excite the oscillation even in a macrospin simulation (see Supplementary Information). The discrepancy between the simulations and analytical model arises from, for example, the fact that the shape magnetic anisotropy field was neglected in the derivation of Eq. (8). An analytical theory including the shape magnetic anisotropy field can be developed; however, this derivation is highly complex. Therefore, we summarize the details of this analytical theory including the shape magnetic anisotropy field in the Supplementary Information, and report only the calculated results of the current–frequency relation here. The black broken lines in Fig. 6 are the results obtained from the theory including the shape magnetic anisotropy field. In the low-current–density region, no oscillation is observed, which indicates that a finite current density is necessary to excite the oscillation. We also note that the results agree well with the estimation from Eq. (8) indicated by the orange broken lines. Again, this agreement demonstrates the validity of Eq. (8), except in the small-current region.

## Conclusions

In summary, we performed micromagnetic simulations for an FM/NM/FM metallic trilayer with strong bilinear and biquadratic interlayer exchange couplings. The bilinear interlayer exchange coupling was found to enhance the oscillation frequency, whereas the biquadratic interlayer exchange coupling provided frequency tunability. A high oscillation frequency of 576 GHz and a wide frequency tunability of 61 GHz/(10^11^ A/m^2^) were obtained by controlling the strengths of the interlayer exchange couplings. An analytical theory based on the macrospin model was also developed, which exhibited good quantitative agreement with the micromagnetic simulations and explained, for example, the linear dependence of the oscillation frequency on the current density. These results indicate that STOs with strong interlayer exchange couplings are promising oscillators for millimeter-wave oscillator applications.

## Methods

### Micromagnetic simulation

In the micromagnetic simulation, each FM is divided into square prisms with dimensions of 2 × 2 × 2 nm^3^. In this work, excluding the investigations on the dependence of the oscillation frequency on $\alpha$ and/or $M$, we use $M=50$ kAm^−1^, $A=0.36\times {10}^{11}$ Jm^−1^, and $\alpha =0.05$ for the free layer while $M=1450$ kAm^−1^, $A=1.00\times {10}^{11}$ Jm^-1^, and $\alpha =0.10$ for the reference layer, where the exchange stiffness $A$ relates to the exchange field ${\mathbf{H}}_{\mathrm{ex}}$ via ${\mathbf{H}}_{\mathrm{ex}}=\left(2A/M\right){\nabla }^{2}{\mathbf{m}}_{i}$. We also use the gyromagnetic ratio $\gamma =1.76\times {10}^{11}$ rad s^−1^ T^-1^. It is assumed here that the current density is homogeneous and independent of the magnetization state. We note that the small saturation magnetization for the free layer assumed in NiCu implies a rich Cu composition, which results in a low transition temperature^48^ and thus is not the most suitable for practical purposes. Low magnetization materials need to be investigated in the future. For example, GaMnAs may satisfy low saturation magnetization as well as a small damping constant, although the low saturation magnetization of 40 kA/m is currently found only at low temperatures^56^. Low magnetization possibly decreases interlayer exchange couplings because the overlapping of spin wavefunctions is small. However, enhancement of interlayer exchange coupling using an ultrathin ferromagnetic insertion layer between FM layer and NM layer has been reported^57^. It is because the ultrathin ferromagnetic insertion layer can improve the overlapping of spin wavefunctions. Therefore, the interlayer exchange couplings can be controlled by the ultrathin ferromagnetic insertion layer while maintaining low magnetization in the FM free layer because the volume of ultrathin ferromagnetic insertion layer is much smaller than that of FM free layer. Using a low magnetization material will contribute to suppressing the enhancement of energy dissipation, although the dissipation typically increases with increasing oscillation frequency. This is because, while the frequency is roughly proportional to the magnitude of the magnetic field, the dissipation is proportional to the product of the magnetic field and saturation magnetization. As shown in the Results, the critical current density necessary to induce the oscillation in the present system is approximately $0.3\times {10}^{11}$ A/m^2^, and the current density exciting an oscillation with a frequency of 100 GHz is of the order of ${10}^{11}$ A/m^2^. These are experimentally available values. Further, the spin current excites the spin-transfer torque acting on the free layer is generated in the reference layer and vice versa. Therefore, it is possible to keep the spin polarization of the spin-transfer torque acting on the free layer high, although low-magnetization materials, such as the NiCu used in the free layer, may be considered to have lower spin polarization. This is because the CoFeB used as the reference layer can have high spin polarization.

The spin polarization $p$ determines the strength of the spin-transfer torque. The spin polarization $p=0.35$ are assumed to be common for two ferromagnets. Equation (1) is solved using the 4th-order Runge–Kutta scheme^58^, where the time increment is essentially $\Delta t=1\times {10}^{-5}$ ns. However, we use $1\times {10}^{-6}$ ns when the frequency is sufficiently high.

## Supplementary Information

Supplementary Information 1.

Supplementary Video 1.

Supplementary Video 2.

## Data Availability

The datasets used and/or analyzed during the current study available from the corresponding author on reasonable request.
