# Supplementary material for: Ultra-wide-band millimeter-wave generator using spin torque oscillator with strong interlayer exchange couplings
Source: Sci Rep. 2022 Jul 19;12:10849. doi: 10.1038/s41598-022-15014-y (PMC9296563; doi:10.1038/s41598-022-15014-y)
Supplement: Supplementary file 1 — Supplementary Information 1. [file 41598_2022_15014_MOESM1_ESM.pdf]

Ultra-wide-band millimeter-wave generator using spin torque oscillator with strong interlayer exchange couplings

Yuichiro Kurokawa[1], Keisuke Yamada[2], Tomohiro Taniguchi[3], Shu Horiike[1], Terumitsu Takana[1], and Hiromi Yuasa[1]

[1]Graduate School and Faculty of Information Science and Electrical Engineering, Kyushu University, Fukuoka 819-0395, Japan
[2]Department of Chemistry and Biomolecular Science, Faculty of Engineering, Gifu University, Gifu 501-1193 Japan
[3]National Institute of Advanced Industrial Science and Technology (AIST), Research Center for Emerging Computing Technologies, Tsukuba, Ibaraki 305-8568, Japan

ABSTRACT

In this Supplementary Information, we develop an analytical approach to evaluate the current-frequency relation of the spin-torque oscillator (STO) studied in the main text. In Sec. 1, we discuss the current-frequency relation in an axially symmetric system and show that the oscillation frequency of the magnetization is proportional to the current. We note that the current-frequency relation in the present STO is well explained by that of the axially symmetric system, even though the STO does not, strictly speaking, have the symmetry. In Sec. 2, we develop an analytical theory, taking into account full magnetic anisotropies, to derive the current-frequency relation. Although the formula is complicated compared with the axially symmetric system, the theory shows good agreement with the numerical simulations shown in the main text. A small amplitude oscillation excited in the reference layer is discussed in Sec. 3.

1 Approximated theory and current-frequency relation

In this section, we study the current-frequency relation of the axially symmetric spin-torque oscillator (STO), and discuss the reason why a simplified relation, i.e., a linear relation between the frequency and current, is obtained in the present STO described in the main text, even though the STO does not have such a simplified symmetry.

1.1 Introduction of the LLG equation

As mentioned in the main text, a macrospin simulation well reproduces the current-frequency relation obtained by the micromagnetic simulation. The Landau-Lifshitz-Gilbert (LLG) equation in the macrospin limit studied in the main text is

$$\frac{\mathrm{d}\mathbf{m}}{\mathrm{d}t} = -\gamma \mathbf{m} \times \mathbf{H} - \gamma H_{\mathrm{s}} \mathbf{m} \times (\mathbf{p} \times \mathbf{m}) + \alpha \mathbf{m} \times \frac{\mathrm{d}\mathbf{m}}{\mathrm{d}t}, \tag{S.1}$$

where γ and α are the gyromagnetic ratio and the Gilbert damping constant, respectively. The unit vectors pointing in the free and reference layers are denoted as $\mathbf{m}$ and $\mathbf{p}$, respectively. The spin torque strength H_{s} is

$$H_{\mathrm{s}} = \frac{\hbar p j}{2eMd}, \tag{S.2}$$

where p are the spin polarization of the current density j, whereas M and d are the saturation magnetization and thickness of the free layer, respectively. The positive current is defined as the electrons flowing from the free to reference layer, i.e., the spin-transfer torque generated by the positive current forces the magnetization of the free layer to become antiparallel to $\mathbf{p}$. Note that the Landé g-factor g, gyromagnetic ratio γ, Bohr magneton μ_{B}, and reduced Planck constant $\hbar$ are related as $g\mu_{\mathrm{B}} = \gamma\hbar$. The magnetic field is given by

$$\mathbf{H} = \begin{pmatrix} h_{\mathrm{bl}} + h_{\mathrm{bq}} m_x \\ 0 \\ -4\pi M m_z \end{pmatrix}, \tag{S.3}$$

where $h_{\rm bl} = J_1/(Md)$ and $h_{\rm bq} = 2J_2/(Md)$ originate from the bilinear and biquadratic couplings between the free and reference layers, whereas $-4\pi M$ is the shape magnetic anisotropy field of the free layer. In this Supplementary Information, we use the following parameters, $M = 50$ emu/cm^3, $\gamma = 1.76 \times 10^7$ rad/(Oe s), $\alpha = 0.05$, $p = 0.35$, $d = 2$ nm, $J_1 = 0.6$ erg/cm^2, and $J_2 = -0.6$ erg/cm^2. We emphasize that J_2, as well as $h_{\rm bq}$, is negative.

We should note that the magnetization dynamics is unchanged even if we add a term proportional to $\mathbf{m}$ to the magnetic field $\mathbf{H}$ because the field in the LLG equation appears in the form of $\mathbf{m} \times \mathbf{H}$, and $\mathbf{m} \times \mathbf{m} = \mathbf{0}$. Therefore, instead of Eq. (S.3), we define the magnetic field as

$$\mathbf{H} = \begin{pmatrix} h_{\rm bl} - (|h_{\rm bq}| - 4\pi M) m_x \\ 4\pi M m_y \\ 0 \end{pmatrix}. \tag{S.4}$$

Note here that $|h_{\rm bq}|$ is 12 T whereas $4\pi M$ is 628 Oe for the present parameters, i.e., $-(|h_{\rm bq}| - 4\pi M)$ is negative. Hence, the free layer has an easy axis along the y direction and a hard axis along the x direction. The field $h_{\rm bl}$ can be regarded as an external magnetic field applied along the hard-axis direction. In addition, we remind the readers that the magnetization $\mathbf{p}$ in the reference layer also points to the hard-axis (x) direction.

1.2 Relation to previous work and redefining magnetic field for this Supplementary Information

We emphasize that the present system is mathematically similar to a magnetic multilayer studied in previous works[1–13], where the free layer is in-plane magnetized whereas the reference layer has a perpendicular magnetization. The structure was proposed to achieve a fast magnetization switching[1,3] and excite a large-amplitude magnetization oscillation[2,4–6,8,11,12], which can be, for example, applied to recording system in hard-disk drive[7,9,10,13]. According to these previous works, in this Supplementary Information, we use a coordinate in which the hard axis, as well as the magnetization in the reference layer, is along the z direction whereas the in-plane easy axis of the free layer points to the x direction. Therefore, the magnetic field in this Supplementary Information is defined as

$$\mathbf{H} = \begin{pmatrix} H_{\rm K} m_x \\ 0 \\ H_z - 4\pi M \bar{N}_z m_z \end{pmatrix}, \tag{S.5}$$

where $H_{\rm K}$ is the in-plane magnetic anisotropy field whereas $4\pi M \bar{N}_z$ is the perpendicular magnetic anisotropy field. The effective demagnetization coefficient $\bar{N}_z$ is defined as $4\pi M \bar{N}_z = 4\pi M N_z - H_{\rm K\perp}$, where $H_{\rm K\perp}$ is, for example, an interfacial perpendicular magnetic anisotropy field[14–16]. In the present system, $4\pi M \bar{N}_z > 0$, indicating that the z axis is the hard axis. Note that $H_{\rm K}$ and $-4\pi M \bar{N}_z$ in Eq. (S.5) correspond to $4\pi M$ and $-(|h_{\rm bq}| - 4\pi M)$ in Eq. (S.4), whereas the external magnetic field H_z in Eq. (S.5) corresponds to $h_{\rm bl}$ in Eq. (S.4). We assume that $|H_z|$ is smaller than $4\pi M \bar{N}_z$ so that the z axis is still the hard axis even in the presence of the external field H_z. The magnetization in the reference layer points to the z direction as $\mathbf{p} = +\mathbf{e}_z$.

1.3 Axially symmetric limit

Using the values of the parameters, we notice that the in-plane and out-of-plane magnetic anisotropy fields are $H_{\rm K} \simeq 1.6$ kOe and $4\pi M \bar{N}_z \simeq 12$ T, respectively, i.e., $H_{\rm K}/(4\pi M \bar{N}_z) \simeq 0.01$. Therefore, in this section, let us neglect the in-plane anisotropy field. The magnetic field is then approximated as

$$\mathbf{H} \simeq \begin{pmatrix} 0 \\ 0 \\ H_z - 4\pi M \bar{N}_z m_z \end{pmatrix}, \tag{S.6}$$

i.e., only the z component is finite. We remind the readers that the magnetization in the reference layer also points to the z direction, $\mathbf{p} = +\mathbf{e}_z$. Therefore, the STO has the axial symmetry around the z axis.

The magnetization direction in the absence of the current is determined in order to minimize the magnetic energy density $E = -M \int d\mathbf{m} = -M H m_z + [(4\pi M^2 \bar{N}_z)/2] m_z^2$, and is given by

$$m_{0z} = \frac{H_z}{4\pi M \bar{N}_z}. \tag{S.7}$$

The auto-oscillation is excited when the spin-transfer torque balances with the damping torque. Therefore, the condition to excite the auto-oscillation is

$$H_{\rm s} \mathbf{m} \times (\mathbf{p} \times \mathbf{m}) + \alpha \mathbf{m} \times (\mathbf{m} \times \mathbf{H}) = \mathbf{0}, \tag{S.8}$$

where we neglect the terms proportional to $\alpha H_{\rm s}$ because, as shown below, $H_{\rm s}$ is at least on the first order of α for the auto-oscillation state, and the damping constant α is usually small[17]. We note that Eq. (S.8) can be easily solved due to the axial symmetry. We notice that m_z is constant in the auto-oscillation state. The current density required to excite an auto-oscillation with a certain value of m_z is given by

$$j(m_z) = \frac{2\alpha eMd}{\hbar p}\left(H_z - 4\pi M \bar{N}_z m_z\right). \tag{S.9}$$

Notice that $\lim_{m_z \to m_{0z}} j(m_z) = 0$, indicating that an infinitesimal current can excite an auto-oscillation. In other words, a critical current density necessary to excite the oscillation is zero.

The oscillation frequency of the auto-oscillation is given by

$$f(m_z) = \frac{\gamma}{2\pi}\left(H_z - 4\pi M \bar{N}_z m_z\right). \tag{S.10}$$

We can see that the current-frequency relation is obtained by using m_z as a parameter. Using Eq. (S.9), we find

$$f = \frac{\gamma \hbar p j}{4\pi \alpha eMd}, \tag{S.11}$$

i.e., the frequency is proportional to the current, which well explains the results shown in the main text.

1.4 Summary of this section

In this Section 1, we first point out that the present STO is mathematically identical to the STO studied in the previous works, where the free and reference layers are magnetized along the in-plane and out-of-plane directions, respectively. Therefore, we rewrite the LLG equation in the form was that used in the previous studies. Using the fact that the in-plane magnetic anisotropy field is negligibly small, i.e., smaller than the perpendicular magnetic anisotropy field, and we can then consider the axially symmetric case where a simplified current-frequency relation, used in the main text, is obtained.

One might be interested in how the in-plane magnetic anisotropy field affects the magnetization oscillation, although the simplified formula obtained in this section works well to explain the results of the numerical simulations in the main text. In next section, we develop an analytical approach to derive the current-frequency formula in the present STO by taking into account the in-plane magnetic anisotropy field.

2 Theoretical analysis on current-frequency relation

In this section, we show the theoretical method to estimate the current-frequency relation of the STO. As mentioned above, the present STO is mathematically identical to the STO with an in-plane easy axis along the x direction and a perpendicular demagnetization field along the z direction. Therefore, we use the model where the magnetic field is given by Eq. (S.5). We remind the readers that the in-plane magnetic anisotropy field was neglected in Sec. 1, whereas it is explicitly included in this section.

2.1 Condition to excite auto-oscillation

In general, Eq. (S.8) cannot be used directly to derive the current-frequency relation because $\mathbf{H}$ is not parallel to $\mathbf{p}$, depending on the magnetization direction. Therefore, we use a relaxed condition, where averages of the spin-transfer and damping torques are balanced. Accordingly, an auto-oscillation of the magnetization is induced when a condition,

$$\oint {\rm d}t \frac{{\rm d}E}{{\rm d}t} = 0, \tag{S.12}$$

is satisfied[18,19], where $E = -M\int {\rm d}\mathbf{m}\cdot\mathbf{H}$ is the magnetic energy density. The range of the time integral is equal to the period of the auto-oscillation. Neglecting the higher-order terms of the small damping constant α, Eq. (S.12) can be rewritten as

$$\mathscr{W}_\alpha + \mathscr{W}_{\rm s} = 0, \tag{S.13}$$

where $\mathscr{W}_\alpha$ and $\mathscr{W}_{\rm s}$ are the energy dissipation and the work done by the spin-transfer torque given by

$$\mathscr{W}_\alpha = -\alpha\gamma M \oint {\rm d}t \left[\mathbf{H}^2 - (\mathbf{m}\cdot\mathbf{H})^2\right], \tag{S.14}$$

$$\mathscr{W}_{\rm s} = \gamma M \oint {\rm d}t H_{\rm s}\left[\mathbf{p}\cdot\mathbf{H} - (\mathbf{m}\cdot\mathbf{p})(\mathbf{m}\cdot\mathbf{H})\right]. \tag{S.15}$$

Strictly speaking, the solution of the LLG equation should be applied to Eqs. (S.14) and (S.15). It is, however, difficult to obtain an exact solution of the LLG equation; in other words, if we could somehow solve the LLG equation exactly, the following analysis would be unnecessary. An approximation to perform the integrals in Eqs. (S.14) and (S.15) is to substitute the solution of the Landau-Lifshitz (LL) equation,

$$\frac{\mathrm{d}\mathbf{m}}{\mathrm{d}t} = -\gamma \mathbf{m} \times \mathbf{H}, \tag{S.16}$$

for that of the LLG equation. Note that the dynamical trajectory described by the solution of the LL equation is a constant energy curve of E, where the value of E is determined to satisfy Eq. (S.13). In fact, the energy averaged over the precession period of the auto-oscillation should be kept constant to excite an sustainable oscillation, and therefore, the oscillation trajectory should be close to the constant energy curve of E. The current-frequency relation is obtained as

1. Substituting a certain value for E in Eq. (S.12), the value of the current density j is determined to satisfy the equation. The value of the current density $j(E)$ is the current necessary to excite the auto-oscillation on the constant energy curve of E.

2. The oscillation frequency $f(E)$ of the magnetization is the inverse of the period for the precession around the constant energy curve of E.

In other words, regarding E as a parameter, the current density $j(E)$ to excite the constant energy curve of E and the frequency $f(E)$ for the precession of the curve are simultaneously determined. From Eq. (S.13), the explicit form of $j(E)$ is given by

$$j(E) = \frac{2\alpha eMd}{\hbar p} \frac{\oint \mathrm{d}t \left[\mathbf{H}^2 - (\mathbf{m}\cdot\mathbf{H})^2\right]}{\oint \mathrm{d}t \left[\mathbf{p}\cdot\mathbf{H} - (\mathbf{m}\cdot\mathbf{p})(\mathbf{m}\cdot\mathbf{H})\right]}. \tag{S.17}$$

We remind the readers that this approach is an extension of the axially symmetric case studied in Sec. 1. As can be seen in Eqs. (S.9) and (S.10), the current-frequency relation in the axially symmetric system is obtained by regarding m_z, which is constant in the auto-oscillation state, as a parameter determining the oscillation state. In general, however, none of the components of $\mathbf{m}$ in the Cartesian coordinate is constant in the auto-oscillation state. Therefore, we prefer to the energy density E as a parameter to derive the current-frequency relation in general cases.

2.2 Energy density

As mentioned in the last section, the trajectory of the auto-oscillation is approximately described by the constant energy curve of E. Therefore, it is necessary to clarify the minimum, saddle points, and maxima of the energy density. The energy density in the present system is given by

$$E = -MH_z m_z - \frac{MH_\mathrm{K}}{2} m_x^2 + \frac{4\pi M^2 \bar{N}_z}{2}. \tag{S.18}$$

The minimum of E is given by

$$E_\mathrm{min} = -M \frac{H_z^2 + H_\mathrm{K}(H_\mathrm{K} + 4\pi M\bar{N}_z)}{2(H_\mathrm{K} + 4\pi M\bar{N}_z)}, \tag{S.19}$$

corresponding to the magnetization directions of

$$\mathbf{m}_{0\pm} = \begin{pmatrix} \pm m_{0x} \\ 0 \\ m_{0z} \end{pmatrix}, \qquad m_{0x} = \sqrt{1 - m_{0z}^2}, \qquad m_{0z} = \frac{H_z}{H_\mathrm{K} + 4\pi M\bar{N}_z}. \tag{S.20}$$

Note that $\mathbf{m}_{0+(-)}$ locates in the positive (negative) x region. In the following, we assume that $|H_z| < H_\mathrm{K} + 4\pi M\bar{N}_z$ to guarantee that the perpendicular (z) direction be unstable.

The saddle points of the energy density locate at

$$\mathbf{m}_{\mathrm{d}\pm} = \begin{pmatrix} 0 \\ \pm m_{\mathrm{d}y} \\ m_{\mathrm{d}z} \end{pmatrix}, \qquad m_{\mathrm{d}y} = \sqrt{1 - m_{\mathrm{d}z}^2} \qquad m_{\mathrm{d}z} = \frac{H_z}{4\pi M\bar{N}_z}, \tag{S.21}$$

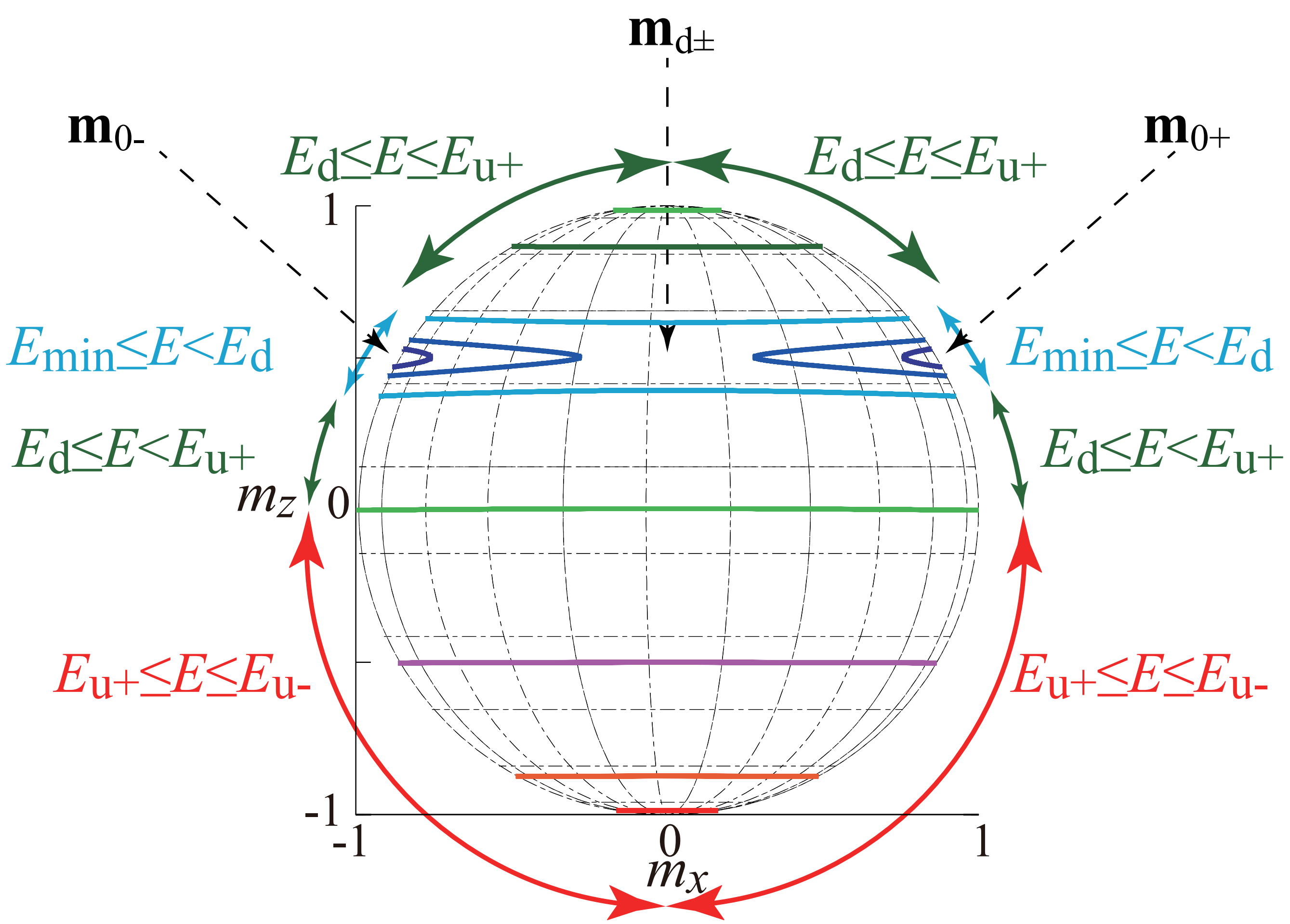

Figure S 1. Constant energy curves of E in the present STO.

corresponding to the saddle-point energy density given by

$$E_d = -\frac{MH_z^2}{8\pi M\bar{N}_z}. \tag{S.22}$$

The unstable states, corresponding to the local maxima of the energy density, locate at $\mathbf{m} = \pm\mathbf{e}_z$, where the corresponding energy densities are

$$E_{u\pm} = \mp MH_z + \frac{4\pi M^2\bar{N}_z}{2}. \tag{S.23}$$

For convention, we also introduce

$$E_u^S = \min\left[E_{u+}, E_{u-}\right], \qquad E_u^L = \max\left[E_{u+}, E_{u-}\right]. \tag{S.24}$$

For the field H_z in the positive (negative) z direction, E_u^S and E_u^L are $E_{u+(-)}$ and $E_{u-(+)}$, respectively. We remind the readers that H_z is positive for the parameters used in the main text.

Figure S1 shows examples of the constant energy curves in the present system. Since the LLG (or LL) equation conserves the norm of the magnetization as $|\mathbf{m}| = 1$, the magnetization dynamics is described by a trajectory on a unit sphere. Accordingly, the constant energy curves are also described on the unit sphere. The constant energy curves for $E_{min} \leq E < E_d$ are the trajectories around $\mathbf{m}_{0\pm}$. In the following, we call this energy region as the stable region. The auto-oscillation in the stable region is the in-plane precession around $\mathbf{m}_{0\pm}$. On the other hand, we call the energy region for $E_d \leq E < E_u^L$ as the unstable region. The constant energy curves of E in the unstable region describe the trajectories around the z axis, as shown in Fig. S1. Hence, the auto-oscillation around the z axis is excited. In the following calculations, the unstable region is further divided

into two regions, where the energy densities are in the range of $E_{\rm d} \le E < E_{\rm u}^{\rm S}$ and $E_{\rm u}^{\rm S} \le E \le E_{\rm u}^{\rm L}$. For the present system having the positive field $H_z > 0$, $E_{\rm u}^{\rm S} = E_{\rm u+}$ and $E_{\rm u}^{\rm L} = E_{\rm u-}$, the energy region of $E_{\rm d} \le E < E_{\rm u}^{\rm S}$ appears in both the positive and negative z regions, whereas the energy region of $E_{\rm u}^{\rm S} \le E \le E_{\rm u}^{\rm L}$ appears only in the negative z region.

It is useful to introduce the following dimensionless quantities,

$$h_z = \frac{H_z}{H_{\rm K} + 4\pi M \bar{N}_z}, \qquad h_{\rm K} = \frac{H_{\rm K}}{H_{\rm K} + 4\pi M \bar{N}_z}, \qquad \varepsilon = \frac{E}{M(H_{\rm K} + 4\pi M \bar{N}_z)}. \tag{S.25}$$

In terms of the dimensionless energy density ε, $E_{\rm min}$, $E_{\rm d}$, and $E_{\rm u\pm}$ are given by

$$\varepsilon_{\rm min} = -\frac{h_{\rm K} + h_z^2}{2}, \qquad \varepsilon_{\rm d} = -\frac{h_z^2}{2(1-h_{\rm K})}, \qquad \varepsilon_{\rm u\pm} = \mp h_z + \frac{1-h_{\rm K}}{2}. \tag{S.26}$$

For the sake of convention, we also introduce $\varepsilon_{\rm u}^{\rm S}$ and $\varepsilon_{\rm u}^{\rm L}$, corresponding to $E_{\rm u}^{\rm S}$ and $E_{\rm u}^{\rm L}$, as

$$\varepsilon_{\rm u}^{\rm S} = \min\left[\varepsilon_{\rm u+}, \varepsilon_{\rm u-}\right], \qquad \varepsilon_{\rm u}^{\rm L} = \max\left[\varepsilon_{\rm u+}, \varepsilon_{\rm u-}\right]. \tag{S.27}$$

Using Eq. (S.18) and the conservation law of the magnetization norm, $|\mathbf{m}| = 1$, the z component of Eq. (S.16) becomes

$$\frac{{\rm d}m_z}{\sqrt{m_z^2 - \frac{2h_z}{1-h_{\rm K}} m_z - \frac{2\varepsilon}{1-h_{\rm K}}}\sqrt{-m_z^2 + 2h_z m_z + 2\varepsilon + h_{\rm K}}} = \gamma\sqrt{1-h_{\rm K}}\,(H_{\rm K} + 4\pi M \bar{N}_z)\,{\rm d}t. \tag{S.28}$$

Note that the denominator of Eq. (S.28) has four roots given by

$$\frac{h_z + \sqrt{h_z^2 + 2(1-h_{\rm K})\varepsilon}}{1-h_{\rm K}}, \qquad \frac{h_z - \sqrt{h_z^2 + 2(1-h_{\rm K})\varepsilon}}{1-h_{\rm K}}, \tag{S.29}$$

$$h_z + \sqrt{h_{\rm K} + h_z^2 + 2\varepsilon}, \qquad h_z - \sqrt{h_{\rm K} + h_z^2 + 2\varepsilon}. \tag{S.30}$$

The physical meanings of the four roots are as follows.

- The roots $m_z = h_z \pm \sqrt{h_{\rm K} + h_z^2 + 2\varepsilon}$ correspond to the points at which the constant energy curve of E crosses a plane of $m_y = 0$.

- The roots $m_z = [h_z \pm \sqrt{h_z^2 + 2(1-h_{\rm K})\varepsilon}]/(1-h_{\rm K})$ correspond to the points at which the constant energy curve of E crosses a plane of $m_x = 0$.

These roots have the following properties, depending on the value of the energy density ε.

- In the energy range of $\varepsilon_{\rm min} \le \varepsilon < \varepsilon_{\rm d}$, the constant energy curves cross the roots $m_z = h_z \pm \sqrt{h_{\rm K} + h_z^2 + 2\varepsilon}$. The time necessary to move between these two points corresponds to the half of the oscillation period. On the other hand, the constant energy curves in this energy region do not cross the line of $m_x = 0$, as can be seen as the blue lines in Fig. S1, and therefore, the roots are complex numbers.

- In the energy range of $\varepsilon_{\rm d} \le \varepsilon < \varepsilon_{\rm u}^{\rm S}$, the four roots are real numbers and their magnitudes are smaller than 1. The roots $h_z + \sqrt{h_{\rm K} + h_z^2 + 2\varepsilon}$ and $[h_z + \sqrt{h_z^2 + 2(1-h_{\rm K})\varepsilon}]/(1-h_{\rm K})$ locate above the energetically stable region, whereas the roots $h_z - \sqrt{h_{\rm K} + h_z^2 + 2\varepsilon}$ and $[h_z - \sqrt{h_z^2 + 2(1-h_{\rm K})\varepsilon}]/(1-h_{\rm K})$ locate below that. The time necessary to move between these two points corresponds to a quarter of the oscillation period.

- In the energy range of $\varepsilon_{\rm u}^{\rm S} \le \varepsilon \le \varepsilon_{\rm u}^{\rm L}$, the four roots are real numbers. This energy region locates around the negative z direction for the positive field $h_z > 0$; in this case, the magnitudes of the roots $h_z + \sqrt{h_{\rm K} + h_z^2 + 2\varepsilon}$ and $[h_z + \sqrt{h_z^2 + 2(1-h_{\rm K})\varepsilon}]/(1-h_{\rm K})$ are larger than 1, whereas those of the roots $h_z - \sqrt{h_{\rm K} + h_z^2 + 2\varepsilon}$ and $[h_z - \sqrt{h_z^2 + 2(1-h_{\rm K})\varepsilon}]/(1-h_{\rm K})$ are smaller than 1. For a negative field, h_z, on the other hand, the magnitudes of the roots $h_z + \sqrt{h_{\rm K} + h_z^2 + 2\varepsilon}$ and $[h_z + \sqrt{h_z^2 + 2(1-h_{\rm K})\varepsilon}]/(1-h_{\rm K})$ are smaller than 1, whereas those of the roots $h_z - \sqrt{h_{\rm K} + h_z^2 + 2\varepsilon}$ and $[h_z - \sqrt{h_z^2 + 2(1-h_{\rm K})\varepsilon}]/(1-h_{\rm K})$ are larger than 1.

2.3 Solutions of the LL equation in the stable region

The solution of the LL equation in the stable region, $\varepsilon_{\min} \le \varepsilon < \varepsilon_{\rm d}$, describes the oscillation of the magnetization around the stable states $\mathbf{m}_{0\pm}$. In this energy region, two roots, $h_z \pm \sqrt{h_{\rm K} + h_z^2 + 2\varepsilon}$, are real numbers, whereas the other roots, $[h_z \pm \sqrt{h_z^2 + 2(1-h_{\rm K})\varepsilon}]/(1-h_{\rm K})$, are complex numbers. Introducing the parameters

$$a = h_z + \sqrt{h_{\rm K} + h_z^2 + 2\varepsilon}, \qquad b = h_z - \sqrt{h_{\rm K} + h_z^2 + 2\varepsilon}, \tag{S.31}$$

$$b_1 = \frac{h_z}{1-h_{\rm K}}, \qquad a_1 = \sqrt{-\frac{h_z^2 + 2(1-h_{\rm K})\varepsilon}{(1-h_{\rm K})^2}}, \tag{S.32}$$

Eq. (S.28) becomes

$$\frac{{\rm d}m_z}{\sqrt{(a-m_z)(m_z-b)[(m_z-b_1)^2+a_1^2]}} = \gamma\sqrt{1-h_{\rm K}}\,(H_{\rm K} + 4\pi M \bar{N}_z)\,{\rm d}t. \tag{S.33}$$

The solution of $\mathbf{m}$ is obtained by integrating Eq. (S.33) in the region of $[m_z, a]$. Then, it is useful to introduce two parameters A and B and new variable u as

$$A = \sqrt{(a-b_1)^2 + a_1^2}, \qquad B = \sqrt{(b-b_1)^2 + a_1^2}, \tag{S.34}$$

$$\sqrt{1-u^2} = \frac{-(a-m_z)B + (m_z-b)A}{(a-m_z)B + (m_z-b)A}. \tag{S.35}$$

The relation between u and m_z is solved as

$$m_z = \frac{aB(1+\sqrt{1-u^2}) + bA(1-\sqrt{1-u^2})}{A+B-(A-B)\sqrt{1-u^2}}. \tag{S.36}$$

Then, the left-hand side of Eq. (S.33) becomes

$$\int_{m_z}^{a} \frac{{\rm d}m_z}{\sqrt{(a-m_z)(m_z-b)[(m_z-b_1)^2+a_1^2]}} = \frac{1}{\sqrt{AB}}\int_0^u \frac{{\rm d}u}{\sqrt{(1-u^2)(1-k^2u^2)}}, \tag{S.37}$$

where we introduce the modulus k of the elliptic integral as

$$k = \sqrt{\frac{(a-b)^2 - (A-B)^2}{4AB}}. \tag{S.38}$$

Therefore, introducing a parameter Ω as

$$\Omega = \gamma\sqrt{AB}\sqrt{1-h_{\rm K}}\,(H_{\rm K} + 4\pi M \bar{N}_z), \tag{S.39}$$

the solution of the LL equation in the stable region is given by

$$m_x = \pm\sqrt{\frac{a^2-b^2}{a^2B^2-b^2A^2}}\,\frac{2AB\,{\rm dn}(\Omega t, k)}{A+B-(A-B){\rm cn}(\Omega t, k)}, \tag{S.40}$$

$$m_y = \mp\sqrt{\frac{AB(a^2-b^2-A^2+B^2)}{a^2B^2-b^2A^2}}\,\frac{(a-b){\rm sn}(\Omega t, k)}{A+B-(A-B){\rm cn}(\Omega t, k)}, \tag{S.41}$$

$$m_z = \frac{bA + aB - (bA - aB){\rm cn}(\Omega t, k)}{A+B-(A-B){\rm cn}(\Omega t, k)}, \tag{S.42}$$

where sn, cn, and dn are the Jacobi elliptic functions. The double sign $\pm$ is $+$ $(-)$ for the oscillation around the $\mathbf{m}_{0+(-)}$. Note that the oscillation frequency of the STO is related to Ω via

$$f(E) = \frac{\Omega}{4\mathsf{K}(k)}, \tag{S.43}$$

where $\mathsf{K}(k) = \int_0^1 {\rm d}t/\sqrt{(1-t^2)(1-k^2t^2)}$ is the complete elliptic integral of the first kind.

2.4 Solutions of the LL equation in the unstable region ($\varepsilon_{\rm d} \le \varepsilon < \varepsilon_{\rm u}^{\rm S}$)

In this section, we show the solution for $\varepsilon_{\rm d} \le \varepsilon < \varepsilon_{\rm u}^{\rm S}$, i.e., the energy density is larger than the saddle point energy density $E_{\rm d}$ and smaller than $\min[E_{\rm u+}, E_{\rm u-}]$. The four roots in Eq. (S.28),

$$a = h_z + \sqrt{h_{\rm K} + h_z^2 + 2\varepsilon}, \qquad b = \frac{h_z + \sqrt{h_z^2 + 2(1-h_{\rm K})\varepsilon}}{1-h_{\rm K}}, \tag{S.44}$$

$$c = \frac{h_z - \sqrt{h_z^2 + 2(1-h_{\rm K})\varepsilon}}{1-h_{\rm K}}, \qquad d = h_z - \sqrt{h_{\rm K} + h_z^2 + 2\varepsilon}, \tag{S.45}$$

are real numbers, and $a > b > 0$ and $d < c < 0$. Note that the symbols a, b, c, and d are used to represent the four routes in descending order. Since the magnitude relation between the four roots depends on the energy ε, we mention the relation between (a,b,c,d) and the four roots in each energy region.

As mentioned around Fig. S1, there are two regions corresponding to the energy range of $\varepsilon_{\rm d} \le \varepsilon < \varepsilon_{\rm u}^{\rm S}$; one locates above the stable region whereas the other locates below the stable region. For a while, let us consider the former unstable region. In this case, we introduce a new variable u as

$$u = \sqrt{\frac{(b-d)(a-m_z)}{(a-b)(m_z-d)}}. \tag{S.46}$$

Then, the left-hand side of Eq. (S.33) becomes

$$\int_{m_z}^{a} \frac{{\rm d}m_z}{\sqrt{(a-m_z)(m_z-b)(m_z-c)(m_z-d)}} = \frac{2}{\sqrt{(a-c)(b-d)}} \int_0^u \frac{{\rm d}u}{\sqrt{(1-u^2)(1-k^2u^2)}}, \tag{S.47}$$

where the modulus k in the present case is

$$k = \sqrt{\frac{(a-b)(c-d)}{(a-c)(b-d)}}. \tag{S.48}$$

Therefore, introducing Ω as

$$\Omega = \gamma \frac{\sqrt{(a-c)(b-d)}}{2} \sqrt{1-h_{\rm K}}\,(H_{\rm K} + 4\pi M \bar{N}_z)\,, \tag{S.49}$$

we find that the solution of Eq. (S.16) becomes

$$m_x = \sqrt{\frac{(a-b)(a-c)(a+d)}{ab(c-d)-cd(a-b)}} \frac{(b-d){\rm cn}(\Omega t,k){\rm dn}(\Omega t,k)}{(b-d)+(a-b){\rm sn}^2(\Omega t,k)}, \tag{S.50}$$

$$m_y = -\sqrt{\frac{(a-b)(b+c)(b-d)}{ab(c-d)-cd(a-b)}} \frac{(a-d){\rm sn}(\Omega t,k)}{(b-d)+(a-b){\rm sn}^2(\Omega t,k)}, \tag{S.51}$$

$$m_z = \frac{a(b-d)+d(a-b){\rm sn}^2(\Omega t,k)}{(b-d)+(a-b){\rm sn}^2(\Omega t,k)}. \tag{S.52}$$

On the other hand, the solution of Eq. (S.16) in the unstable region located lower with respect to the stable region is given by

$$m_x = \sqrt{\frac{(b-d)(c-d)(a+d)}{ab(c-d)-cd(a-b)}} \frac{(a-c){\rm cn}(\Omega t,k){\rm dn}(\Omega t,k)}{(a-c)+(c-d){\rm sn}^2(\Omega t,k)}, \tag{S.53}$$

$$m_y = \sqrt{\frac{(c-d)(b+c)(a-c)}{ab(c-d)-cd(a-b)}} \frac{(a-d){\rm sn}(\Omega t,k)}{(a-c)+(c-d){\rm sn}^2(\Omega t,k)}, \tag{S.54}$$

$$m_z = \frac{d(a-c)+a(c-d){\rm sn}^2(\Omega t,k)}{(a-c)+(c-d){\rm sn}^2(\Omega t,k)}. \tag{S.55}$$

2.5 Solutions of the LL equation in the unstable region ($\varepsilon_{\rm u}^{\rm S} \le \varepsilon \le \varepsilon_{\rm u}^{\rm L}$)

The unstable region corresponding to the energy density of $\varepsilon_{\rm u}^{\rm S} \le \varepsilon \le \varepsilon_{\rm u}^{\rm L}$ exists close to $\mathbf{m} = -(+)\mathbf{e}_z$ when H_z is positive (negative); see also Fig. S1. Note that the magnitude relation between the four roots depends on H_z in this case. Performing calculations similar to those studied in the last section, we find that, the solution of the LL equation in this energy region is given by

- When H_z is positive,

$$m_x = \sqrt{\frac{(a-d)(c-d)(b+d)}{ab(c-d)+cd(a-b)}}\frac{(a-c)\mathrm{cn}(\Omega t,k)}{(a-c)+(c-d)\mathrm{sn}^2(\Omega t,k)}, \tag{S.56}$$

$$m_y = \sqrt{\frac{(a+c)(a-c)(a-d)(b-d)(c-d)}{ab(c-d)+cd(a-b)}}\frac{\mathrm{sn}(\Omega t,k)\mathrm{dn}(\Omega t,k)}{(a-c)+(c-d)\mathrm{sn}^2(\Omega t,k)}, \tag{S.57}$$

$$m_z = \frac{d(a-c)+a(c-d)\mathrm{sn}^2(\Omega t,k)}{(a-c)+(c-d)\mathrm{sn}^2(\Omega t,k)}, \tag{S.58}$$

where

$$a = \frac{h_z+\sqrt{h_z^2+2(1-h_{\rm K})\varepsilon}}{1-h_{\rm K}}, \qquad b = h_z+\sqrt{h_{\rm K}+h_z^2+2\varepsilon}, \tag{S.59}$$

$$c = \frac{h_z-\sqrt{h_z^2+2(1-h_{\rm K})\varepsilon}}{1-h_{\rm K}}, \qquad d = h_z-\sqrt{h_{\rm K}+h_z^2+2\varepsilon}. \tag{S.60}$$

- When H_z is negative

$$m_x = \sqrt{\frac{(a-b)(a-d)(a+c)}{-ab(c-d)-cd(a-b)}}\frac{(b-d)\mathrm{cn}(\Omega t,k)}{(b-d)+(a-b)\mathrm{sn}^2(\Omega t,k)}, \tag{S.61}$$

$$m_y = -\sqrt{\frac{(a-b)(a-c)(a-d)(b+d)(b-d)}{-ab(c-d)-cd(a-b)}}\frac{\mathrm{sn}(\Omega t,k)\mathrm{dn}(\Omega t,k)}{(b-d)+(a-b)\mathrm{sn}^2(\Omega t,k)} \tag{S.62}$$

$$m_z = \frac{a(b-d)+d(a-b)\mathrm{sn}^2(\Omega t,k)}{(b-d)+(a-b)\mathrm{sn}^2(\Omega t,k)} \tag{S.63}$$

where

$$a = h_z+\sqrt{h_{\rm K}+h_z^2+2\varepsilon}, \qquad b = \frac{h_z+\sqrt{h_z^2+2(1-h_{\rm K})\varepsilon}}{1-h_{\rm K}}, \tag{S.64}$$

$$c = h_z-\sqrt{h_{\rm K}+h_z^2+2\varepsilon}, \qquad d = \frac{h_z-\sqrt{h_z^2+2(1-h_{\rm K})\varepsilon}}{1-h_{\rm K}}. \tag{S.65}$$

The modulus k and the angular frequency Ω are given by

$$k = \sqrt{\frac{(a-b)(c-d)}{(a-c)(b-d)}}, \tag{S.66}$$

$$\Omega = \gamma\frac{\sqrt{(a-c)(b-d)}}{2}\sqrt{1-h_{\rm K}}\,(H_{\rm K}+4\pi M\bar{N}_z)\,. \tag{S.67}$$

We remind the readers that $h_{\rm bl}$ in the main text, corresponding to H_z here, points to the positive z direction.

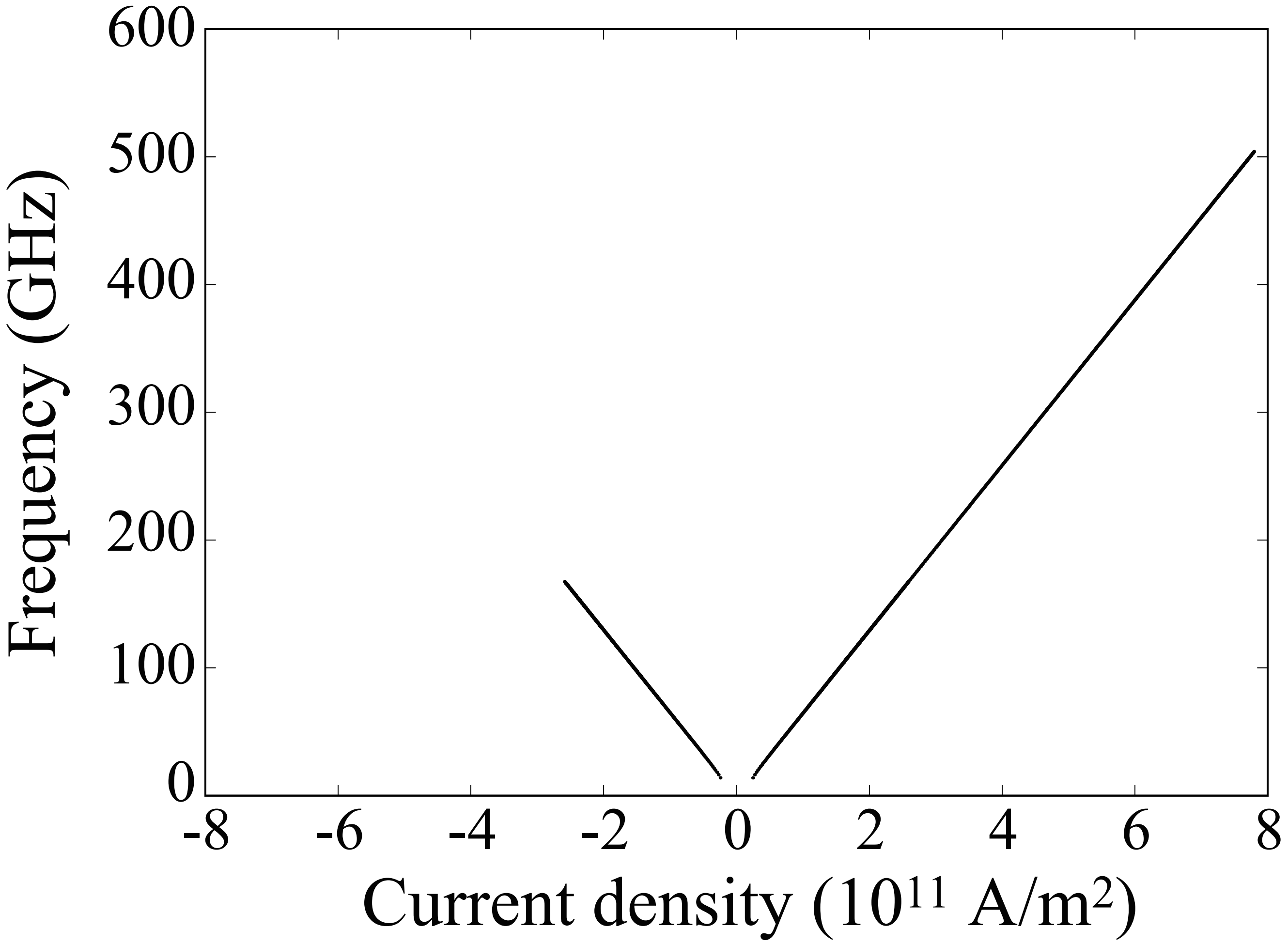

Figure S 2. Current-frequency relation for the auto-oscillation around the *z* axis.

2.6 Current-frequency relation for auto-oscillation in unstable region

Applying Eqs. (S.50)-(S.55) or Eqs. (S.56)-(S.58) to Eqs. (S.14) and (S.15) and solving Eq. (S.13) with respect to the current density, we obtain the current-frequency relation for the auto-oscillation in the energetically unstable region, corresponding to the oscillation around the z axis, shown in Fig. S2. The current density is evaluated from Eq. (S.17), whereas the frequency is given by $f(E) = \Omega/[4\mathsf{K}(k)]$. We remind the readers that the spin-transfer torque due to negative (positive) current moves the magnetization to the positive (negative) z direction. Due to the presence of the positive field ($H_z > 0$), the unstable region in the positive z region is relatively small, as can be observed in Fig. S1. Thus, the magnetization saturates to the positive z direction by relatively small current density, $j \simeq -2.6 \times 10^{11}$ A/m^2, where the oscillation disappears. On the other hand, the oscillation is excited over a wide range of the positive current. This is because the unstable region in the negative z region is relatively large, as shown in Fig. S1.

Readers should keep in mind that the current-frequency relation shown in Fig. S2 is obtained by using the solution of the LL equation, not the LLG equation. Nevertheless, it shows good agreement with the result obtained by the LLG simulation in the negative-current region, guaranteeing the applicability of the present theoretical method; see also Sec. 3. We also emphasize that the frequency is nearly proportional to the current density. This result indicates that the present STO is well approximated by the axially-symmetric system studied in Sec. 1 due to the small in-plane magnetic anisotropy field $[H_{\mathrm{K}}/(4\pi M\bar{N}_z) \ll 1]$, and therefore, Eq. (S.11) well describes the current-frequency relation. Simultaneously, however, be reminded the readers that the approximated formula, Eq. (S.11), cannot explain the existence of the threshold current below which the magnetization is fixed to the in-plane equilibrium state, and thus, the auto-oscillation will not be excited.

2.7 Instability threshold

The analysis developed above assumes the magnetization oscillation on a constant energy curve. However, it has been found in a similar system that an instability threshold exists to excite the oscillation, i.e., the oscillation occurs when the current density exceeds the threshold value, whereas the magnetization saturates to fixed points and does not show oscillation when the current is below the threshold value. For example, in Refs.[10–12], where the ferromagnet is in-plane magnetized as in the present system but the in-plane magnetic anisotropy field is zero and the external magnetic field is tilted from the z axis, a finite current was necessary to excite the magnetization oscillation around the z axis. In this section, let us estimate the threshold value of the present system in the macrospin limit.

As written, when the magnitude of the current density is small, the magnetization moves from the energetically stable state to a fixed point, i.e., the magnetization finally satisfies $\mathrm{d}\mathbf{m}/\mathrm{d}t = \mathbf{0}$. Therefore, we investigate the solution of the magnetization at the fixed point. In terms of the zenith and azimuth angles (θ, φ) defined as $\mathbf{m} = (\sin\theta\cos\varphi, \sin\theta\sin\varphi, \cos\theta)$, the LLG equation (S.1) can be expressed as

$$\frac{\mathrm{d}\theta}{\mathrm{d}t} = -\frac{\gamma}{M\sin\theta}\frac{\partial E}{\partial \varphi} - \gamma H_{\mathrm{s}}\frac{\partial}{\partial\theta}\mathbf{m}\cdot\mathbf{p} - \alpha\sin\theta\frac{\mathrm{d}\varphi}{\mathrm{d}t}, \tag{S.68}$$

$$\sin\theta\frac{\mathrm{d}\varphi}{\mathrm{d}t} = \frac{\gamma}{M}\frac{\partial E}{\partial\theta} - \frac{\gamma H_{\mathrm{s}}}{\sin\theta}\frac{\partial}{\partial\varphi}\mathbf{m}\cdot\mathbf{p} + \alpha\frac{\mathrm{d}\theta}{\mathrm{d}t}. \tag{S.69}$$

The condition $\mathrm{d}\mathbf{m}/\mathrm{d}t = 0$ implies that

$$\left(H_z - H_{\mathrm{K}}\cos\theta\cos^2\varphi - 4\pi M\bar{N}_z\cos\theta\right)\sin\theta = 0, \tag{S.70}$$

$$(-H_{\mathrm{K}}\sin\varphi\cos\varphi + H_{\mathrm{s}})\sin\theta = 0. \tag{S.71}$$

Since we investigate a fixed point near the energetically stable states, which locate close to $\mathbf{m} = \pm\mathbf{e}_x$, i.e., $\theta = \pi/2$, the solution of $\sin\theta = 0$ is out of interest. Therefore, the solution of φ is found as

$$\sin 2\varphi = \frac{2H_{\mathrm{s}}}{H_{\mathrm{K}}}. \tag{S.72}$$

We note that $m_x = \sin\theta\cos\varphi$ and $m_z = \cos\theta$ at the fixed point can be estimated from the following relations,

$$\cos^2\varphi = \frac{1}{2}\left[1 + \sqrt{1 - \left(\frac{2H_{\mathrm{s}}}{H_{\mathrm{K}}}\right)^2}\right], \qquad \cos\theta = \frac{H_z}{H_{\mathrm{K}}\cos^2\varphi + 4\pi M\bar{N}_z}. \tag{S.73}$$

Since $|\sin 2\varphi| < 1$, the fixed point exists when the magnitude of the current density is smaller than a critical value,

$$j_{\mathrm{c}} = \frac{2eMd}{\hbar p}\frac{H_{\mathrm{K}}}{2}. \tag{S.74}$$

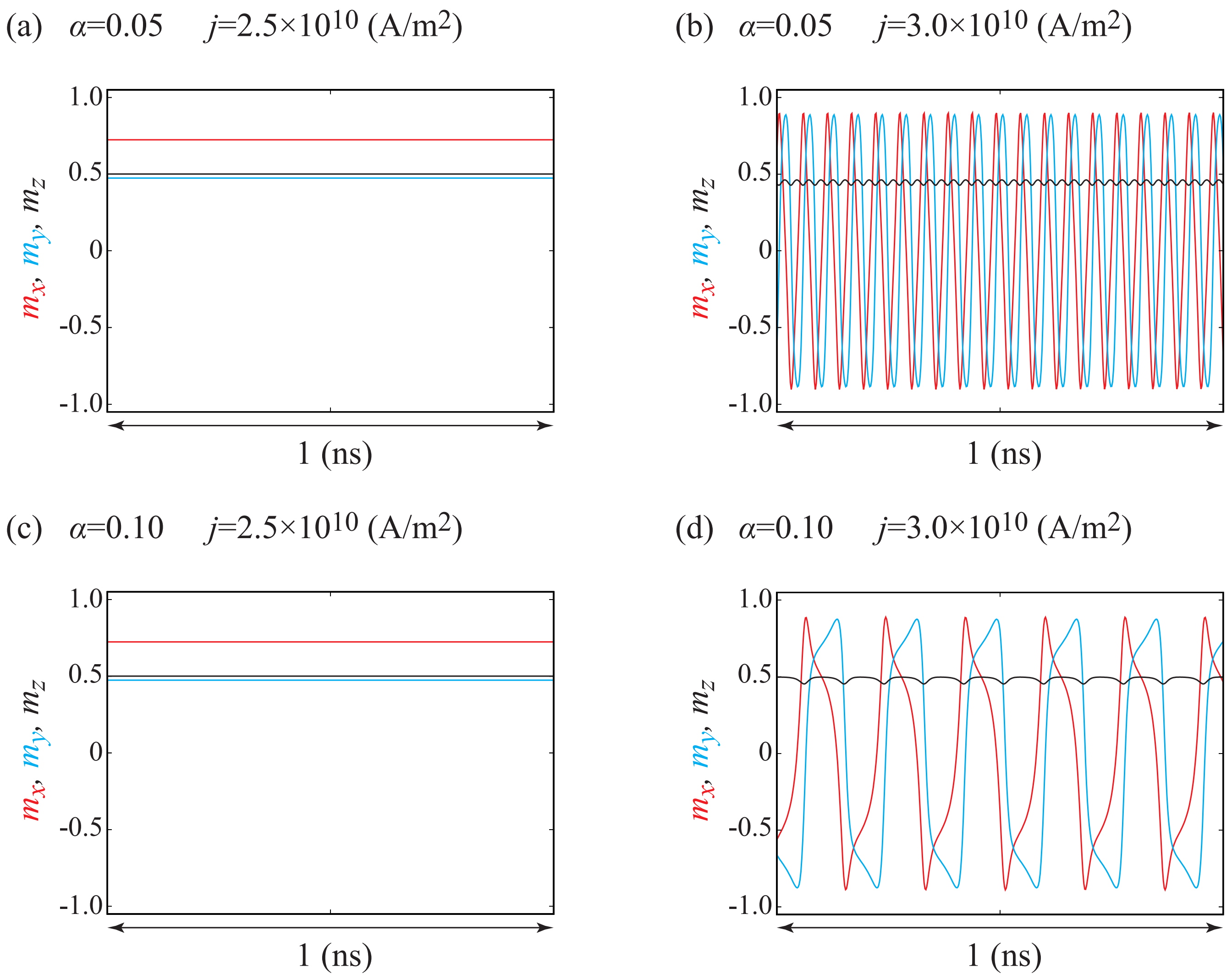

Figure S 3. Steady state solutions of $\mathbf{m} = (m_x, m_y, m_z)$. The damping constant α is 0.05 in (a) and (b), whereas it is 0.10 in (c) and (d). The current density j is 2.5×10^{10} A/m^2 in (a) and (c), whereas it is 3.0×10^{10} A/m^2 in (b) and (d).

We study the validity of Eq. (S.74) by solving Eq. (S.1) numerically. Note that the value of j_c is 2.7×10^{10} A/m^2. Figure S3 summarizes $\mathbf{m}$ in a steady state, The damping constant α is 0.05 in Figs. S3(a) and S3(b), whereas it is 0.10 in Figs. S3(c) and S3(d). The current density j is 2.5×10^{10} A/m^2 in Figs. S3(a) and S3(c), whereas it is 3.0×10^{10} A/m^2 in Figs. S3(b) and S3(d). The magnetization is saturated to a fixed point when j is below j_c, whereas it shows an oscillation when j is larger than j_c. The results also imply that the threshold current density is independent of the value α, which is consistent with Eq. (S.74). The solution of $\mathbf{m}$ below the threshold is also independent of α, as observed in Figs. S3(a) and S3(c). These results indicate the validity of Eq. (S.74). We also note that the oscillation properties, such as the frequency, above the threshold depend on α, as indicated in Figs. S3(b) and S3(d), because the oscillation condition depends on the damping torque through Eq. (S.13). The oscillation trajectory is well described by trigonometric functions when the damping constant is small, as observed in Fig. S3(b) because the magnetization deviates from the energetically stable region and shows approximately axially-symmetric oscillation. On the other hand, when the damping constant is large, the oscillation occurs near the energetically stable region, and the dynamical trajectory becomes relatively complex, as shown in Fig. S3(d), reflecting the fact that the trajectory is described by the elliptic functions, discussed in previous sections.

3 Small amplitude oscillation in reference layer

In the above sections, we assume that the direction of the magnetization in the reference layer is fixed. In reality, however, small-amplitude dynamics are excited in reference layer. In this section, we show that the magnetization oscillation in the positive-current region and discuss that the oscillation frequency cannot be explained by the model developed in the previous sections. We also show that the difference between the numerical simulation and the analytical model for the positive-current region originates from the magnetization oscillation in the reference layer. The asymmetry in the magnetization oscillation in the reference layer with respect to the current direction is explained by the linearized LLG equation.

3.1 Oscillation behaviors in positive current region

Here, we describe the magnetization dynamics behavior in the positive-current region. Figure S4(a) shows the time evolutions of the averaged components of $\mathbf{m}_1$ and $|\mathbf{m}_1|$ for $j = +1.0 \times 10^{11}$ A/m^2. Here, the oscillation frequency is 23 GHz, which is smaller than that induced by negative currents of the same magnitude. We found that, even when the absolute current density was increased, the oscillation frequency in the positive-current region was smaller than that in the negative-current region. As an example, Fig. S4(b) shows the time evolutions of the averaged components of $\mathbf{m}_1$ and the averaged $|\mathbf{m}_1|$ for $j = +2.0 \times 10^{11}$ A/m^2, where the oscillation frequency, 107 GHz, is smaller than that (120 GHz) found in the negative-current region.

Figure S5 summarizes the normalized Fourier spectrum of m_{1y} obtained by micromagnetic simulation. The solid line corresponds to Eq. (S.11) obtained by assuming an axially symmetric system, while the dotted line is identical to that shown in Fig. S2. As mentioned, Eq. (S.11) shows good agreements with both micromagnetic simulation and theory developed in Sec. 2 when the sign of the current is negative, although Eq. (S.11) cannot explain, for example, the existence of the instability threshold. We also remind the readers that Eq. (S.11) predicts a symmetric dependence of the oscillation frequency with respect to the sign of current. However, Fig. S5 indicates that the dependence of the oscillation frequency on the current in micromagnetic simulation is smaller than that predicted by Eq. (S.11) for the positive-current region.

The asymmetric dynamical behavior of the oscillation frequency with respect to the current direction is related to the magnetization dynamics in the reference layer. Figures S6(a) and S6(b) show the time evolutions of m_{2y} and m_{2z} in the reference layer, for (a) $j = -2.0 \times 10^{11}$ A/m^2 and (b) $j = +2.0 \times 10^{11}$ A/m^2. Although the magnetization in the reference layer is approximately fixed with a small-amplitude oscillation for the negative-current case, the oscillation amplitude becomes relatively large for the positive-current case.

The different dynamical behaviors in the reference layer originate from the dipole and interlayer exchange couplings to the free layer. These couplings provide the reference layer with an oscillating magnetic field through magnetization oscillation in the free layer. Additionally, these couplings, as well as the pinning field, simultaneously provide the reference layer with a static (dc) magnetic field. These two contributions induce magnetization oscillation in the reference layer in the same (opposite) direction when the current is positive (negative). Hence, the oscillation amplitude of the magnetization in the reference layer becomes relatively large (small) for a positive (negative) current. The magnetization oscillation in the reference layer also affects that in the free layer through the couplings. Therefore, the oscillation behavior generated by the positive current becomes complex and differs from that generated by a negative current.

3.2 Magnetic field acting on the reference layer and rotation direction of magnetization

Here, we show an analysis on the effect of the oscillating magnetic field generated from the free layer on the magnetization dynamics in the reference layer, which will clarify the dependence of the oscillation amplitude in the reference layer on the current direction. We apply macrospin model to the reference layer. As used in the above sections, we denote the unit vectors

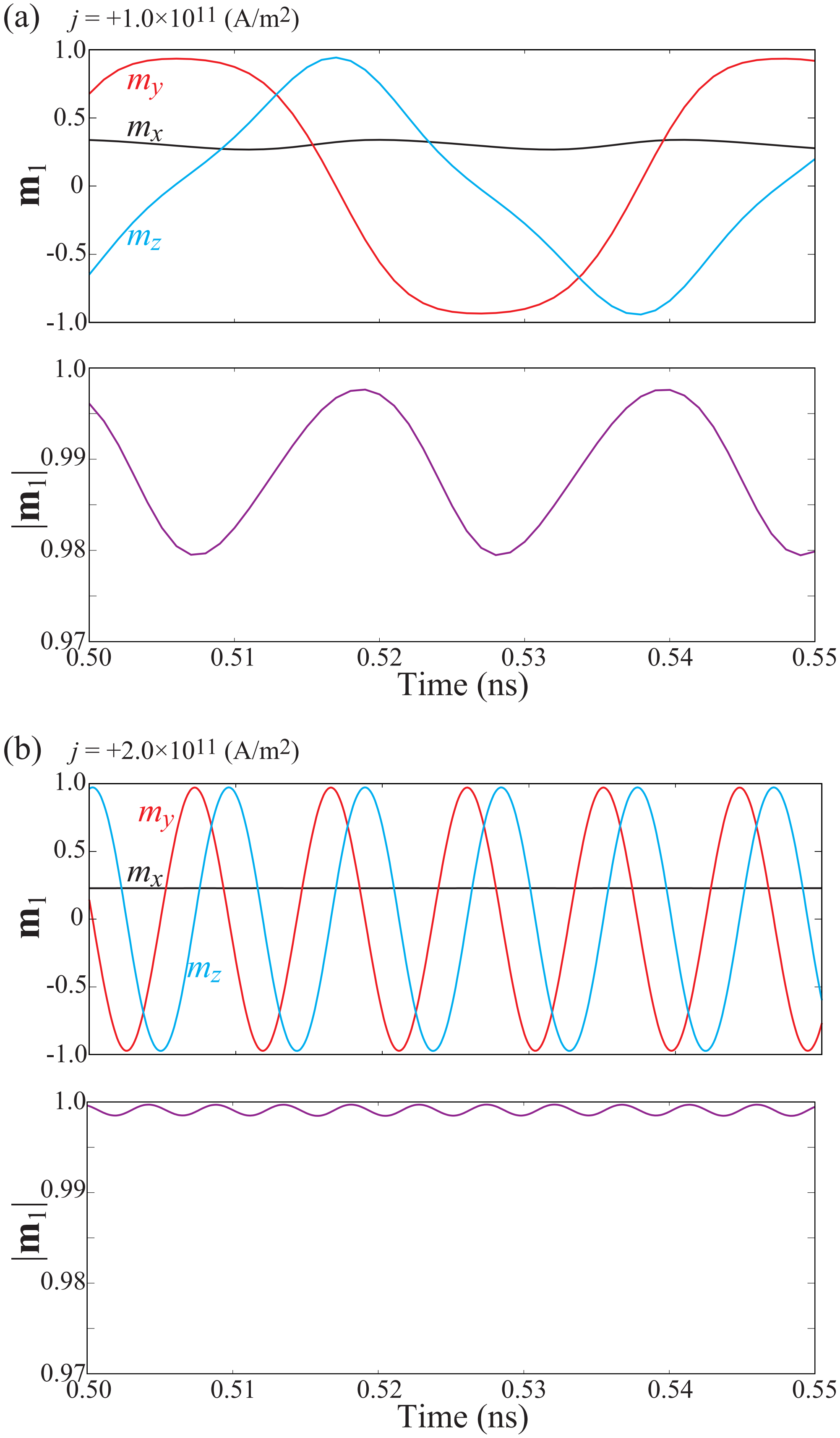

Figure S 4. (a,b) Time evolution of averaged $\mathbf{m}_1$ and its magnitude $|\mathbf{m}_1|$ for $j = +1.0 \times 10^{11}$ and $+2.0 \times 10^{11}$ A/m^2, respectively. The interlayer exchange couplings are $(J_1, J_2) = (0.6, -0.6)$ mJ/m^2.

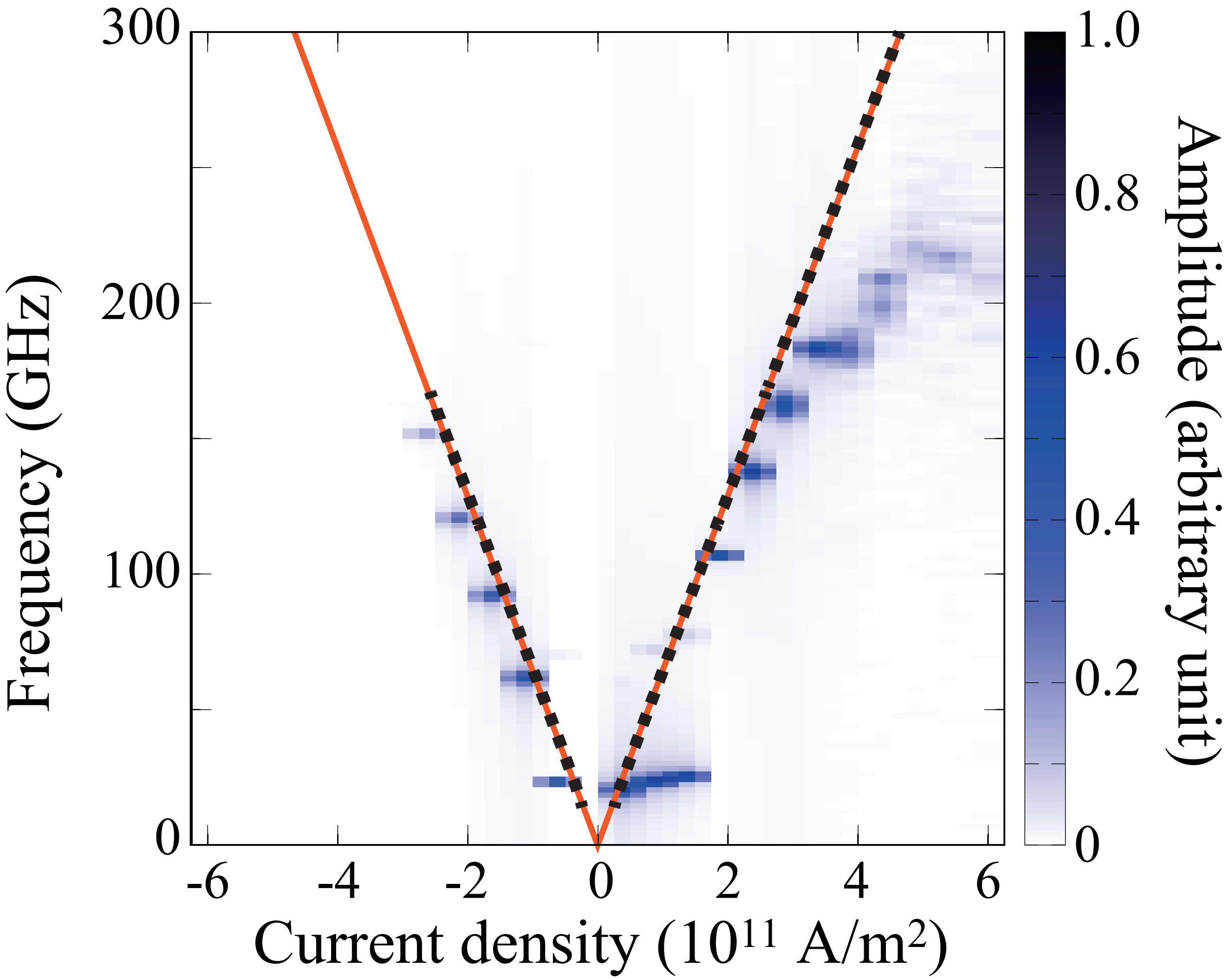

Figure S 5. Normalized Fourier spectrum of m_{1y} obtained by micromagnetic simulation. The orange solid line is estimated from Eq. (S.11), while the black dotted line is identical to that shown in Fig. S2.

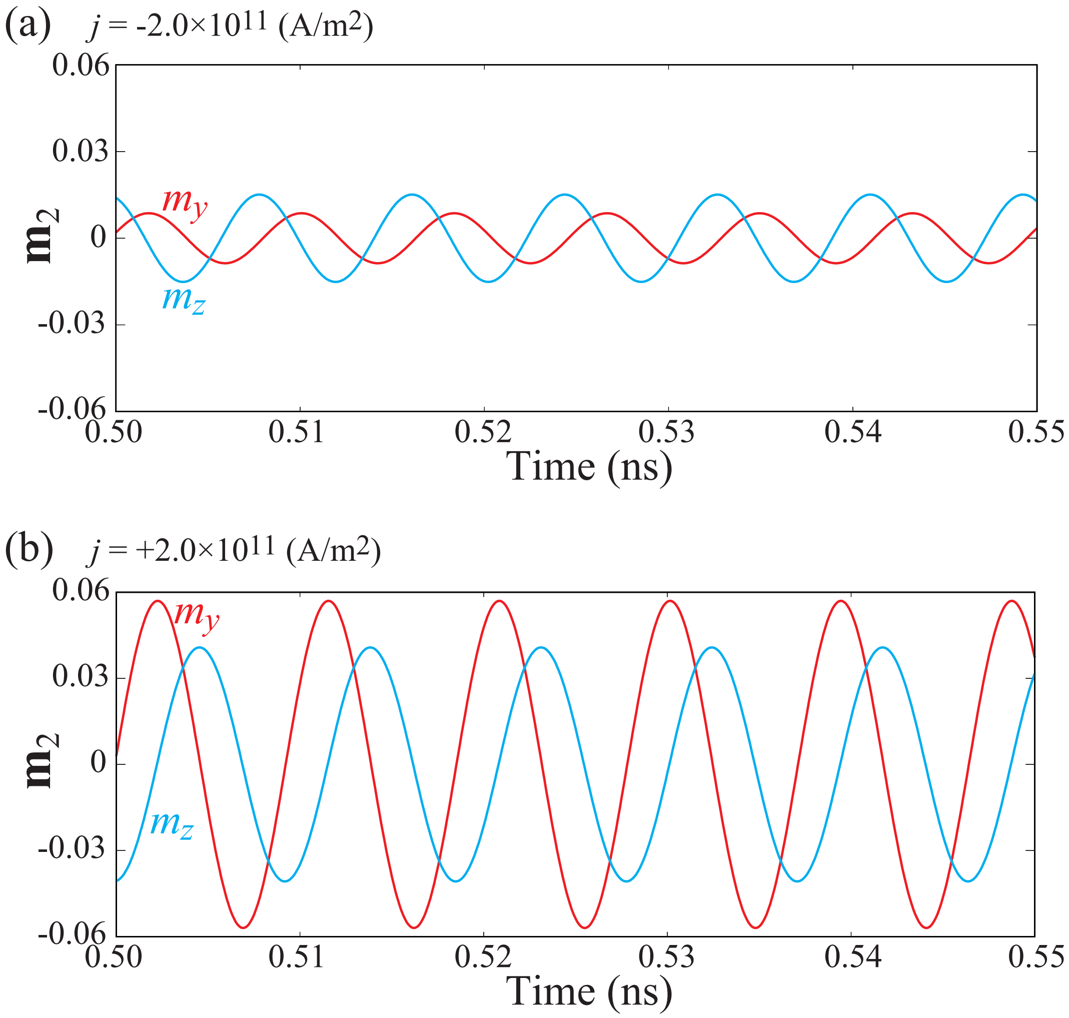

Figure S 6. (a) Time evolution of averaged m_{2y} and m_{2z} for $(J_1, J_2) = (0.6, -0.6)$ mJ/m^2 under (a) $j = -2.0 \times 10^{11}$ and (b) $+2.0 \times 10^{11}$ A/m^2.

pointing in the free and reference layer as $\mathbf{m}$ and $\mathbf{p}$, respectively. The magnetic field acting on the magnetization $\mathbf{p}$ in the reference layer is given by

$$\mathbf{H}_{\rm p} = -\sum_{i=x,y,z} 4\pi M_2 N_i p_i \mathbf{e}_i + H_{J12}\mathbf{m} + H_{J22}(\mathbf{m}\cdot\mathbf{p})\mathbf{m} + \mathbf{H}_{\rm d} + H_{\rm pin}\mathbf{e}_x, \tag{S.75}$$

where N_i $(i=x,y,z)$ is the demagnetization coefficient, whereas $H_{J12} = J_1/(M_2 d_2)$ and $H_{J22} = J_2/(M_2 d_2)$ are the bilinear and biquadratic interlayer exchange coupling fields, respectively. The dipole field from the free layer is denoted as $\mathbf{H}_{\rm d}$, whose value in the macrospin limit has been estimated in, for example. Ref.[20]. The pinning field generated from the pinned layer is denoted as $H_{\rm pin}$. Since the magnetization in the free layer oscillates around the x axis, the magnetic field affecting on $\mathbf{p}$ can be decomposed into dc component along the x direction and ac component in the yz plane as

$$\mathbf{H}_{\rm p} \simeq \begin{pmatrix} H_p - 4\pi M_2 N_x p_x \\ -4\pi M_2 N_y p_y \\ -4\pi M_2 N_z p_z \end{pmatrix} + \mathbf{h}. \tag{S.76}$$

The dc component of the effective magnetic field, H_p is given by

$$H_p \simeq (H_{J12} + H_{J22} m_x - H_{\rm d1}) m_x + H_{\rm pin}, \tag{S.77}$$

where $H_{\rm d1}$ is the magnitude of the dipole field from the free layer[20]. We use the approximation that $\mathbf{m}\cdot\mathbf{p} \simeq m_x$ for the direct component. Note that m_x is approximately determined by Eq. (S.9), where we remind the readers that we used a coordinate in which the z axis in Sec. 1 is parallel to the axis of the oscillation. In addition, we notice that $\mathbf{p}$ is approximately fixed to the positive x direction, i.e., $p_x \simeq 1$. Using these facts, we notice that the dc component of the effective magnetic field, defined as

$$\mathscr{H} = H_p - 4\pi M_2 N_x, \tag{S.78}$$

is typically positive in our system, which means that the torque due to the dc component of the magnetic field induces an oscillation of $\mathbf{p}$ around the x axis in the counterclockwise direction. On the other hand, $\mathbf{h}(\perp \mathbf{e}_x)$ denotes the ac component of the magnetic field acting on $\mathbf{p}$, which is generated from the oscillating components of the interlayer exchange coupling and dipole fields from the free layer. The ac component of the magnetic field, $\mathbf{h}$, induces an oscillation of $\mathbf{p}$ in the same rotation direction as that of $\mathbf{m}$. In the following, we show that the oscillation amplitude of $\mathbf{p}$ becomes large (small) when the rotation direction of the ac magnetic field, i.e., the oscillation direction of $\mathbf{m}$, is in the counterclockwise (clockwise) direction.

3.3 Linearized LLG equation

We solve the LLG equation of the magnetization direction in the reference layer with the assumption that $p_x \simeq 1$. For simplicity, we neglect the spin-transfer torque acting on $\mathbf{p}$ because its magnitude is relatively small compared with, for example, the pinning field. The LLG equation of $\mathbf{p}$ is hence given by

$$\frac{{\rm d}\mathbf{p}}{{\rm d}t} = -\gamma \mathbf{p} \times \mathbf{H}_{\rm p} + \alpha_2 \mathbf{p} \times \frac{{\rm d}\mathbf{p}}{{\rm d}t}. \tag{S.79}$$

Neglecting the higher order terms of p_y and p_z, the linearized LLG equation of $\mathbf{p}$ becomes[11],

$$\frac{1}{\gamma}\frac{\rm d}{{\rm d}t}\begin{pmatrix} p_y \\ p_z \end{pmatrix} + \begin{pmatrix} \alpha_2 H_X & H_Y \\ -H_X & \alpha_2 H_Y \end{pmatrix}\begin{pmatrix} p_y \\ p_z \end{pmatrix} = \begin{pmatrix} h_z \\ -h_y \end{pmatrix}. \tag{S.80}$$

Here, we introduce $H_X = \mathscr{H} + 4\pi M_2(N_z - N_x) - 4\pi M_2(N_z - N_y) = \mathscr{H}$ and $H_Y = H_X = \mathscr{H} + 4\pi M_2(N_z - N_x)$, where we use $N_x = N_y$ because the reference layer is of a cylinder shape. The y and z components of the oscillating magnetic field, $\mathbf{h}$, are denoted as h_y and h_z. We remind the readers that h_y and h_z originate from the interlayer exchange coupling and dipole fields from the free layer. We also note that the magnetization in the free layer, $\mathbf{m}$, oscillates around the x axis with approximately the same amplitudes for to the y and z directions. Therefore, h_y and h_z will be written in the form of

$$h_y = h_{\rm ac}\cos\omega t, \qquad h_z = h_{\rm ac}\sin\omega t, \tag{S.81}$$

where ω is the angular velocity of the oscillation of $\mathbf{m}$, whereas $h_{\rm ac}$ is the amplitude of the oscillating magnetic field. Note that ω is positive (negative) when $\mathbf{m}$ oscillates around the x axis in the counterclockwise (clockwise) direction. Note also that the oscillation in the counterclockwise (clockwise) direction is excited when the current is positive (negative) and therefore, the spin-transfer torque forces the magnetization $\mathbf{m}$ in the free layer to move the negative (positive) x direction.

To solve Eq. (S.80), it is useful to introduce complex numbers, $\eta_y e^{i\omega t}$ and $\eta_z e^{i\omega t}$, instead of h_z and $-h_y$, and investigate the solutions of p_y and p_z in the forms of $p_y = \tilde{p}_y e^{i\omega t}$ and $p_z = \tilde{p}_z e^{i\omega t}$. Neglecting the higher order terms of the small parameters α_2, the solutions of the oscillation amplitudes, $\tilde{p}_y$ and $\tilde{p}_z$, are obtained as

$$\begin{pmatrix} \tilde{p}_y \\ \tilde{p}_z \end{pmatrix} = \frac{1}{\Delta} \begin{pmatrix} -\frac{i\omega}{\gamma} - \alpha_2 H_Y & H_Y \\ -H_X & -\frac{i\omega}{\gamma} - \alpha_2 H_X \end{pmatrix} \begin{pmatrix} \eta_y \\ \eta_z \end{pmatrix}, \tag{S.82}$$

where Δ is given by

$$\Delta = \left(\frac{\omega}{\gamma}\right)^2 - \left(\frac{\omega_r}{\gamma}\right)^2 - i\frac{\omega\Delta\omega}{\gamma^2}. \tag{S.83}$$

The resonance frequency $f_r = \omega_r/(2\pi)$ and the linewidth $\Delta f = \Delta\omega/(2\pi)$ of ferromagnetic resonance (FMR) in the reference layer are, respectively, given by

$$f_r = \frac{\gamma}{2\pi}\sqrt{H_X H_Y}, \qquad \Delta f = \frac{\alpha_2 \gamma}{2\pi}(H_X + H_Y). \tag{S.84}$$

In the complex form, the solution of $p_y = \tilde{p}_y e^{i\omega t}$ is given by

$$p_y = -\frac{\left(\frac{i\omega}{\gamma} + \alpha H_Y\right)\left[\left(\frac{\omega}{\gamma}\right)^2 - \left(\frac{\omega_r}{\gamma}\right)^2 + i\frac{\omega\Delta\omega}{\gamma^2}\right]}{\left[\left(\frac{\omega}{\gamma}\right)^2 - \left(\frac{\omega_r}{\gamma}\right)^2\right]^2 + \left(\frac{\omega\Delta\omega}{\gamma^2}\right)^2}\eta_y e^{i\omega t} + \frac{H_Y\left[\left(\frac{\omega}{\gamma}\right)^2 - \left(\frac{\omega_r}{\gamma}\right)^2 + i\frac{\omega\Delta\omega}{\gamma^2}\right]}{\left[\left(\frac{\omega}{\gamma}\right)^2 - \left(\frac{\omega_r}{\gamma}\right)^2\right]^2 + \left(\frac{\omega\Delta\omega}{\gamma^2}\right)^2}\eta_z e^{i\omega t}. \tag{S.85}$$

Be reminded that $\eta_y e^{i\omega t}$ and $\eta_z e^{i\omega t}$ were introduced to replace $h_z = h_{ac}\sin\omega t$ and $-h_y = -h_{ac}\cos\omega t$. Assuming that $\eta_y = \eta_z = h_{ac}$, we note that $h_z = \mathrm{Im}[\eta_y e^{i\omega t}]$ and $h_y = -\mathrm{Re}[\eta_z e^{i\omega t}]$. Therefore, the solution of p_y in the real space is obtained by evaluating the imaginary (real) part of the solution in the complex form with respect to the input $\eta_y e^{i\omega t}$ ($\eta_z e^{i\omega t}$) as

$$\begin{aligned} p_y =& \mathrm{Im}\left[\frac{-\left(\frac{i\omega}{\gamma} + \alpha H_Y\right)\left[\left(\frac{\omega}{\gamma}\right)^2 - \left(\frac{\omega_r}{\gamma}\right)^2 + i\frac{\omega\Delta\omega}{\gamma^2}\right]}{\left[\left(\frac{\omega}{\gamma}\right)^2 - \left(\frac{\omega_r}{\gamma}\right)^2\right]^2 + \left(\frac{\omega\Delta\omega}{\gamma^2}\right)^2}\eta_y e^{i\omega t}\right] - \mathrm{Re}\left[\frac{H_Y\left[\left(\frac{\omega}{\gamma}\right)^2 - \left(\frac{\omega_r}{\gamma}\right)^2 + i\frac{\omega\Delta\omega}{\gamma^2}\right]}{\left[\left(\frac{\omega}{\gamma}\right)^2 - \left(\frac{\omega_r}{\gamma}\right)^2\right]^2 + \left(\frac{\omega\Delta\omega}{\gamma^2}\right)^2}\eta_z e^{i\omega t}\right] \\ \simeq& -\frac{\left(\frac{\omega}{\gamma} + H_Y\right)\left[\left(\frac{\omega}{\gamma}\right)^2 - \left(\frac{\omega_r}{\gamma}\right)^2\right]}{\left[\left(\frac{\omega}{\gamma}\right)^2 - \left(\frac{\omega_r}{\gamma}\right)^2\right]^2 + \left(\frac{\omega\Delta\omega}{\gamma^2}\right)^2} h_{ac}\cos\omega t + \frac{-\alpha H_Y\left[\left(\frac{\omega}{\gamma}\right)^2 - \left(\frac{\omega_r}{\gamma}\right)^2\right] + \left(\frac{\omega}{\gamma} + H_Y\right)\frac{\omega\Delta\omega}{\gamma^2}}{\left[\left(\frac{\omega}{\gamma}\right)^2 - \left(\frac{\omega_r}{\gamma}\right)^2\right]^2 + \left(\frac{\omega\Delta\omega}{\gamma^2}\right)^2} h_{ac}\sin\omega t. \end{aligned} \tag{S.86}$$

where we neglect the higher order term, proportional to $\alpha H_Y \Delta\omega$, of α_2. Similarly, from the complex form of p_z,

$$p_z = -\frac{H_X\left[\left(\frac{\omega}{\gamma}\right)^2 - \left(\frac{\omega_r}{\gamma}\right)^2 + i\frac{\omega\Delta\omega}{\gamma^2}\right]}{\left[\left(\frac{\omega}{\gamma}\right)^2 - \left(\frac{\omega_r}{\gamma}\right)^2\right]^2 + \left(\frac{\omega\Delta\omega}{\gamma^2}\right)^2}\eta_y e^{i\omega t} - \frac{\left(\frac{i\omega}{\gamma} + \alpha H_X\right)\left[\left(\frac{\omega}{\gamma}\right)^2 - \left(\frac{\omega_r}{\gamma}\right)^2 + i\frac{\omega\Delta\omega}{\gamma^2}\right]}{\left[\left(\frac{\omega}{\gamma}\right)^2 - \left(\frac{\omega_r}{\gamma}\right)^2\right]^2 + \left(\frac{\omega\Delta\omega}{\gamma^2}\right)^2}\eta_z e^{i\omega t}, \tag{S.87}$$

the solution of p_z is obtained as

$$\begin{aligned} p_y =& \mathrm{Im}\left[\frac{-H_X\left[\left(\frac{\omega}{\gamma}\right)^2 - \left(\frac{\omega_r}{\gamma}\right)^2 + i\frac{\omega\Delta\omega}{\gamma^2}\right]}{\left[\left(\frac{\omega}{\gamma}\right)^2 - \left(\frac{\omega_r}{\gamma}\right)^2\right]^2 + \left(\frac{\omega\Delta\omega}{\gamma^2}\right)^2}\eta_y e^{i\omega t}\right] - \mathrm{Re}\left[\frac{-\left(\frac{i\omega}{\gamma} + \alpha H_X\right)\left[\left(\frac{\omega}{\gamma}\right)^2 - \left(\frac{\omega_r}{\gamma}\right)^2 + i\frac{\omega\Delta\omega}{\gamma^2}\right]}{\left[\left(\frac{\omega}{\gamma}\right)^2 - \left(\frac{\omega_r}{\gamma}\right)^2\right]^2 + \left(\frac{\omega\Delta\omega}{\gamma^2}\right)^2}\eta_z e^{i\omega t}\right] \\ \simeq& \frac{\alpha H_X\left[\left(\frac{\omega}{\gamma}\right)^2 - \left(\frac{\omega_r}{\gamma}\right)^2\right] - \left(\frac{\omega}{\gamma} + H_X\right)\frac{\omega\Delta\omega}{\gamma^2}}{\left[\left(\frac{\omega}{\gamma}\right)^2 - \left(\frac{\omega_r}{\gamma}\right)^2\right]^2 + \left(\frac{\omega\Delta\omega}{\gamma^2}\right)^2} h_{ac}\cos\omega t - \frac{\left(\frac{\omega}{\gamma} + H_X\right)\left[\left(\frac{\omega}{\gamma}\right)^2 - \left(\frac{\omega_r}{\gamma}\right)^2\right]}{\left[\left(\frac{\omega}{\gamma}\right)^2 - \left(\frac{\omega_r}{\gamma}\right)^2\right]^2 + \left(\frac{\omega\Delta\omega}{\gamma^2}\right)^2} h_{ac}\sin\omega t. \end{aligned} \tag{S.88}$$

The solutions of p_y and p_z are proportional to $\sin \omega t$ and $-\cos \omega t$ when the resonance condition is satisfied, which indicate that the phase of the magnetization $\mathbf{p}$ shifts 90° from the oscillating magnetic field, $\mathbf{h} = h_{ac} \cos \omega t \mathbf{e}_y + h_{ac} \sin \omega t \mathbf{e}_z$.

In general, however, the angular velocity ω of the oscillating magnetic field is different from ω_r because ω is determined by the oscillation frequency of the magnetization $\mathbf{m}$ in the free layer. Note that the denominators in Eqs. (S.86) and (S.88) are even functions of ω. On the other hand, the numerators of Eqs. (S.86) and (S.88) have odd functions of ω given by $(\omega/\gamma) + H_X$ and $(\omega/\gamma) + H_Y$. According to their definitions, H_X and H_Y are positive in our system. Therefore, the magnitudes of the numerators in Eqs. (S.86) and (S.88), as well as those of p_y and p_z, become relatively large (small) when ω is positive (negative). We should be reminded that ω is positive (negative) when the current is positive (negative). Accordingly, the oscillation amplitude of the magnetization in the reference layer becomes large (small) for the positive (negative) current, which is consistent with the results obtained from the numerical simulation in the main text. We note that the positive signs "+" in the terms $(\omega/\gamma) + H_X$ and $(\omega/\gamma) + H_Y$ originate from the torque $-\gamma \mathbf{p} \times \mathbf{H}_p$ due to the magnetic field $\mathbf{H}_p$ in Eq. (S.79). Therefore, we conclude that the oscillation amplitude of the magnetization $\mathbf{p}$ in the reference layer becomes relatively large (small) when the rotation direction of the magnetization $\mathbf{m}$ in the free layer is the same (opposite) to the direction of the torque due to the dc component of the magnetic field $\mathbf{H}_p$ in the reference layer. The rotation direction of $\mathbf{p}$ is determined by their competition.

When the magnetization $\mathbf{m}$ in the free layer moves close to the yz plane by the spin-transfer torque originated from positive current, the ac component of the oscillating magnetic field acting on $\mathbf{p}$ dominates in $\mathbf{H}_p$. In such a case, nonuniform oscillation can be excited in the reference layer, which also induces nonuniform dynamics in the free layer. Accordingly, the macrospin model does not work well in the positive-current region. On the other hand, the spin-transfer torque induced by negative current moves the magnetization in the free layer close to the x axis. In this case, the ac component of the oscillating magnetic field on $\mathbf{p}$ becomes small. Therefore, the magnetization in the reference layer is approximately fixed to the x direction, and the macrospin model well reproduces the results of micromagnetic simulations.

Acknowledgement

T. Taniguchi is grateful to Takehiko Yorozu for valuable discussions.

References

1. Kent, A. D., Özyilmaz, B. & del Barco, E. Spin-transfer-induced precessional magnetization reversal. *Appl. Phys. Lett.* **84**, 3897 (2004).

2. Lee, K. J. & Redon, O. Analytical investigation of spin-transfer dynamics using a perpendicular-to-plane polarizer. *Appl. Phys. Lett.* **86**, 022505 (2005).

3. Seki, T., Mitani, S., Yakushiji, K. & Takanashi, K. Magnetization reversal by spin-transfer torque in 90° configuration with a perpendicular spin polarizer. *Appl. Phys. Lett.* **89**, 172504 (2006).

4. Houssameddine, D. *et al.* Spin-torque oscillator using a perpendicular polarizer and a planer free layer. *Nat. Mater.* **6**, 447 (2007).

5. Firastrau, I. *et al.* State diagram for spin current-induced magnetization dynamics using a perpendicular polarizer and a planer free layer. *J. Magn. Magn. Mater.* **310**, 2029 (2007).

6. Ebels, U. *et al.* Macrospin description of the perpendicular polarizer-planar free-layer spin-torque oscillator. *Phys. Rev. B* **78**, 024436 (2008).

7. Zhu, J.-G., Zhu, X. & Tang, Y. Microwave Assisted Magnetic Recording. *IEEE Trans. Magn.* **44**, 125 (2008).

8. Silva, T. J. & Keller, M. W. Theory of Thermally Induced Phase Noise in Spin Torque Oscillators for a High-Symmetry Case. *IEEE Trans. Magn.* **46**, 3555 (2010).

9. Bosu, S. *et al.* Reduction of critical current density for out-of-plane mode oscillation in a mag-flip spin torque oscillator suing highly spin-polarized $Co_2Fe(Ga_{0.5}Ge_{0.5})$ spin injection layer. *Appl. Phys. Lett.* **108**, 072403 (2016).

10. Hiramatsu, R. *et al.* Magnetic field angle dependence of out-of-plane precession in spin torque oscillators having an in-plane magnetized free layer and a perpendicularly magnetized reference layer. *Appl. Phys. Express* **9**, 053006 (2016).

11. Taniguchi, T. & Kubota, H. Instability analysis of spin-torque oscillator with an in-plane magnetized free layer and a perpendicularly magnetized pinned layer. *Phys. Rev. B* **93**, 174401 (2016).

12. Taniguchi, T. Crossover between fast and slow excitation of magnetization by spin torque. *Appl. Phys. Express* **9**, 073003 (2016).

13. Bosu, S. *et al.* High frequency out-of-plane oscillation with large cone angle in mag-flip spin torque oscillators for microwave assisted magnetic recording. *Appl. Phys. Lett.* **110**, 142403 (2017).

14. Yakata, S. *et al.* Influence of perpendicular magnetic anisotropy on spin-transfer switching current in CoFeB/MgO/CoFeB magnetic tunnel junctions. *J. Appl. Phys.* **105**, 07D131 (2009).

15. Ikeda, S. *et al.* A perpendicular-anisotropy CoFeB-MgO magnetic tunnel junction. *Nat. Mater.* **9**, 721 (2010).

16. Kubota, H. *et al.* Enhancement of perpendicular magnetic anisotropy in FeB free layers using a thin MgO cap layer. *J. Appl. Phys.* **111**, 07C712 (2012).

17. Oogane, M. *et al.* Magnetic Damping in Ferromagnetic Thin Films. *Jpn. J. Appl. Phys.* **45**, 3889 (2006).

18. Bertotti, G., Mayergoyz, I. & Serpico, C. *Nonlinear Magnetization Dynamics in Nanosystems* (Elsevier, Oxford, 2009).

19. Taniguchi, T. Nonlinear analysis of magnetization dynamics excited by spin Hall effect. *Phys. Rev. B* **91**, 104406 (2015).

20. Taniguchi, T. An analytical computation of magnetic field generated from a cylinder ferromagnet. *J. Magn. Magn. Mater.* **452**, 464 (2018).
